# Past projections of submediterranean oaks unveil future range shifts of vulnerable taxa under climate change

**DOI:** 10.1038/s41598-026-56522-5

**Published:** 2026-06-04

**Authors:** Carlos Vila-Viçosa, Salvador Arenas-Castro, Antoine Guisan, Elif Deniz Ülker, Jean Stephan, Isabel Passos, Francisco M. Vázquez, Rubim Almeida, João Honrado, Cristina García, João Gonçalves

**Affiliations:** 1 BIOPOLIS Program in Genomics, Biodiversity and Land Planning, CIBIO, University of Porto Campus de Vairão, Rua Padre Armando Quintas, 4485-661 Vairão, Portugal; 2 CIBIO (Research Center in Biodiversity and Genetic Resources) - InBIO (Research Network in Biodiversity and Evolutionary Biology), University of Porto. Campus de Vairão, Rua Padre Armando Quintas, 4485-661 Vairão, Portugal; 3 Department of Biology, Faculty of Sciences, University of Porto, Rua do Campo Alegre, s/n, 4169-007 Porto, Portugal; 4 MHNC-UP - Museu de História Natural e da Ciência da Universidade do Porto – Herbário PO, Universidade do Porto, Praça Gomes Teixeira, 4099-002 Porto, Portugal; 5 Área de Ecología, Departamento de Botánica, Ecología y Fisiología Vegetal, Facultad de Ciencias, Universidad de Córdoba, Campus de Rabanales, 14014 Córdoba, Spain; 6 Department of Ecology and Evolution, Faculty of Biology and Medicine, Université de Lausanne, UNIL Sorge Le Biophore, 1015 Lausanne, Switzerland; 7 Functional Ecology Lab. Department of Biology (Ecology Section), Hacettepe University, Ankara, Turkey; 8 Nature Conservation Centre, Çiğdem Mahallesi 1594, Sokak No: 3, 06530 Çankaya/Ankara, Turkey; 9 Laboratory of Georesources, Geosciences and Environment, Faculty of Sciences 2, Lebanese University, Fanar, Lebanon; 10 CERNAS-IPCB – Research Centre for Natural Resources, Environment and Society, Instituto Politécnico de Castelo Branco, Castelo Branco, Portugal; 11 Centre of Studies in Geography and Spatial Planning, Department of Geography and Tourism, University of Coimbra, Colégio de São Jerónimo, 3004-530 Coimbra, Portugal; 12 CICYTEX - Centro de Investigaciones Científicas y Tecnológicas de Extremadura; Agricultural Research Centre, Finca La Orden, Ctra. A-V, Km372, 06187 ValdesequeraGuadajira, Badajoz, Spain; 13 Department of Biological Sciences. Bourne Building, Office 3-29B, Royal Holloway University of London (RHUL) Egham, Surrey, TW20 EX UK; 14 proMetheus—Research Unit in Materials, Energy and Environment for Sustainability, Instituto Politécnico de Viana do Castelo (IPVC), Avenida do Atlântico, No. 644, 4900-348 Viana Do Castelo, Portugal

**Keywords:** Biodiversity conservation, Biogeography, Climate change, Forecast, Hindcast, Mediterranean forests, Quercus, Species distribution models, Climate sciences, Ecology, Ecology, Environmental sciences

## Abstract

Submediterranean marcescent oak forests form a climatic ecotone highly exposed to increasing aridity across the Mediterranean Basin. Understanding how these vulnerable taxa were affected by past climatic shifts can help contextualize their sensitivity to ongoing changes. Here we used ensemble Species Distribution Models (SDMs) to infer the distribution dynamics of eight marcescent oak species from the Heinrich Stadial (~ 17 ka) to the present, and to explore their potential future trajectories for 2070 and 2100 under three SSP scenarios. Models calibrated with 12,450 filtered occurrences and high-resolution paleoclimate and CHELSA datasets performed well (AUC > 0.97), with precipitation and temperature seasonality emerging as key predictors. Hindcasts revealed contrasting east–west Quaternary histories, including episodes of expansion, contraction and partial stability linked to abrupt climate transitions such as the Heinrich Stadial and Younger Dryas. Future projections indicate widespread northward shifts and substantial suitability losses, especially under SSP5-8.5, with pronounced impacts on narrowly distributed taxa. By comparing past-to-present and present-to-future range shifts, we identify temporal coherence in species responses, showing that taxa with strong historical fluctuations tend to exhibit larger projected changes. Integrating past range dynamics provides an essential ecological baseline to interpret species-specific sensitivity and regional asymmetries. Our results refine the identification of potential climatic refugia and high-risk zones, offering a framework to prioritize conservation strategies for transitional oak forests in a rapidly warming Mediterranean Basin.

**Supplementary Information:**

The online version contains supplementary material available at 10.1038/s41598-026-56522-5.

## Introduction

Climate change threatens the high plant diversity of the Mediterranean Basin, one of the world’s major biodiversity hotspots^1,2^. Projections suggest severe biodiversity loss by 2100 due to declining summer rainfall, rising temperatures, and increased droughts^3^. Increased aridity is expected to strongly affect marcescent oak forests, which form a narrow submediterranean ecotonal belt between temperate deciduous forests and Mediterranean evergreen woodlands, and depend on moderate summer rainfall and mild winters^4,5^. Marcescent oaks, which retain dead leaves in winter, are highly sensitive to changing precipitation cycles and droughts, but regional impacts across the East and West Mediterranean remain uncertain^4^. This uncertainty hinders robust forecasts for this key phylogeographic hotspot^6–9^.

Oaks are keystone woody plants that dominate climax forests and scrub stages across diverse edaphic and bioclimatic conditions^10–14^. Drought and winter cold further constrain the climatic envelope of these forests, limiting their distribution across southern European and Mediterranean landscapes^13–15^. Marcescent white oaks belong to the white oak lineage (Section Quercus), a major clade within the genus Quercus, which includes most Eurasian and North American deciduous and marcescent oaks. Within this lineage, marcescent species form a distinct submediterranean forest ecotone between temperate deciduous forests and Mediterranean evergreen woodlands, especially in areas where Atlantic climatic influence moderates summer drought^4,11,16,17^. Their taxonomic diversity increases towards southern Europe, North Africa and the Near East, reflecting complex paleobiogeographic histories^18,19^.

Many submediterranean oaks are narrowly distributed, threatened and highly sensitive to declining water availability and increased drought^4,20^ . Thus, this subset of oaks and the forests they form emerges as a priority for efforts to mitigate plant loss in the Mediterranean Basin^4,21^. However, it remains unclear whether eastern and western Mediterranean species will respond differently to future climate change. This uncertainty is strongly linked to the well-documented east–west asymmetry in Late-Quaternary Mediterranean climates. Paleoclimate records indicate that western Mediterranean regions experienced comparatively cooler and wetter conditions during key intervals such as the Bølling–Allerød and Holocene, favouring the persistence of mesic forest taxa^22^. In contrast, eastern Mediterranean areas were marked by stronger continentality and pronounced summer aridity throughout the Heinrich Stadial, Younger Dryas and Early Holocene, consistent with regional palaeoclimatic syntheses and recent hydroclimatic reconstructions based on multiproxy and model-based evidence^23–26^. These divergent climatic trajectories shaped contrasting refugial dynamics, expansion–contraction patterns and long-term stability for Mediterranean oaks^4,7,27–29^. Recognising this asymmetry is therefore essential for evaluating whether eastern and western submediterranean species will respond differently to future climatic stress, as already suggested for western marcescent oak systems under future climate scenarios^30,31^.

Species Distribution Models (SDMs) have been widely used to infer these past dynamics and to assess the impacts of environmental change on biodiversity^9,32^. Combining hindcasts with forecasts can therefore provide an integrated perspective on species’ range behaviour, offering insights into climatic thresholds, historical suitability dynamics and potential responses under ongoing warming^8,29,33–35^. However, few studies have explicitly quantified whether past-to-present range dynamics are associated with projected future shifts within the same modelling framework. Here, we address this gap by comparing hindcasted and forecasted suitability patterns for submediterranean oaks and by testing whether historical range dynamics help contextualise future responses. This gap is particularly relevant in the Mediterranean, where future projections indicate decreasing precipitation, intensifying summer heat and increased ecosystem vulnerability^3^, potentially exacerbating extreme drought episodes^36^. Yet, the specific impacts on submediterranean oaks remain unclear. In spite of the fact that future climatic regimes may include non-analogue combinations of temperature and precipitation relative to Late-Quaternary conditions^8,33,35^, hindcasts here serve not to improve predictive accuracy but to provide ecological context on species’ sensitivity to climatic variability.

To bridge this gap, we assess the distribution shifts of eight narrowly distributed marcescent oaks (*Quercus boissieri, Q. canariensis, Q. faginea, Q. infectoria, Q. kotschyana, Q. lusitanica, Q. pyrenaica* and *Q. vulcanica*) under increasing aridity. We hypothesize that the climatic factors that historically shaped the distribution of submediterranean oaks may also help elucidate how these species respond to abrupt climatic worsening. Because species experienced different magnitudes of contraction and expansion across Late-Quaternary phases of climatic deterioration and amelioration, we expect that these long-term differences in climatic sensitivity may help contextualise the scale of future shifts under ongoing climate change. We also consider that the contrasting Quaternary climates of eastern and western Mediterranean regions could lead to regionally distinct patterns of future suitability. Together, these considerations provide a coherent ecological rationale for comparing past-to-present and present-to-future dynamics across species and regions. In this study, we used SDMs to hindcast and forecast species’ climatic suitability across six Late-Quaternary periods, comprising Heinrich Stadial 1, the Bølling–Allerød, the Younger Dryas, the Early Holocene, the Mid Holocene and the Late Holocene, and under projected conditions for 2070 and 2100. These intervals encompass phases of climatic deterioration (Heinrich Stadial 1 and Younger Dryas) and climatic amelioration (Bølling–Allerød and the Holocene), allowing species responses to contrasting climatic constraints to be compared. Future projections follow IPCC scenario availability and represent standard mid- and late-century benchmarks commonly used to inform biodiversity conservation and adaptation planning. The SSPs applied bracket a wide spectrum of plausible socio-economic pathways, from moderate mitigation to high-emissions trajectories^37^. We also tested potential east–west contrasts and discuss the implications of these patterns for Mediterranean oak forest conservation.

## Results

### Models performance

Overall, model performance was high (Supplementary Table S1). TSS scores were highest for narrow-range oaks such as *Q. kotschyana* (1.00) and Q. lusitanica (0.96), followed by *Q. vulcanica* (0.91) and *Q. canariensis* (0.90). The lowest TSS values were for Q. infectoria (0.84) and Q. faginea (0.82), still indicating good predictive accuracy. Consistently, these two species also had the lowest AUC (0.97), while all others reached 0.99. Sensitivity and specificity remained above 0.86 for all taxa, confirming reliable prediction of presences and absences.

### Variables contribution

**Fig. 1:**
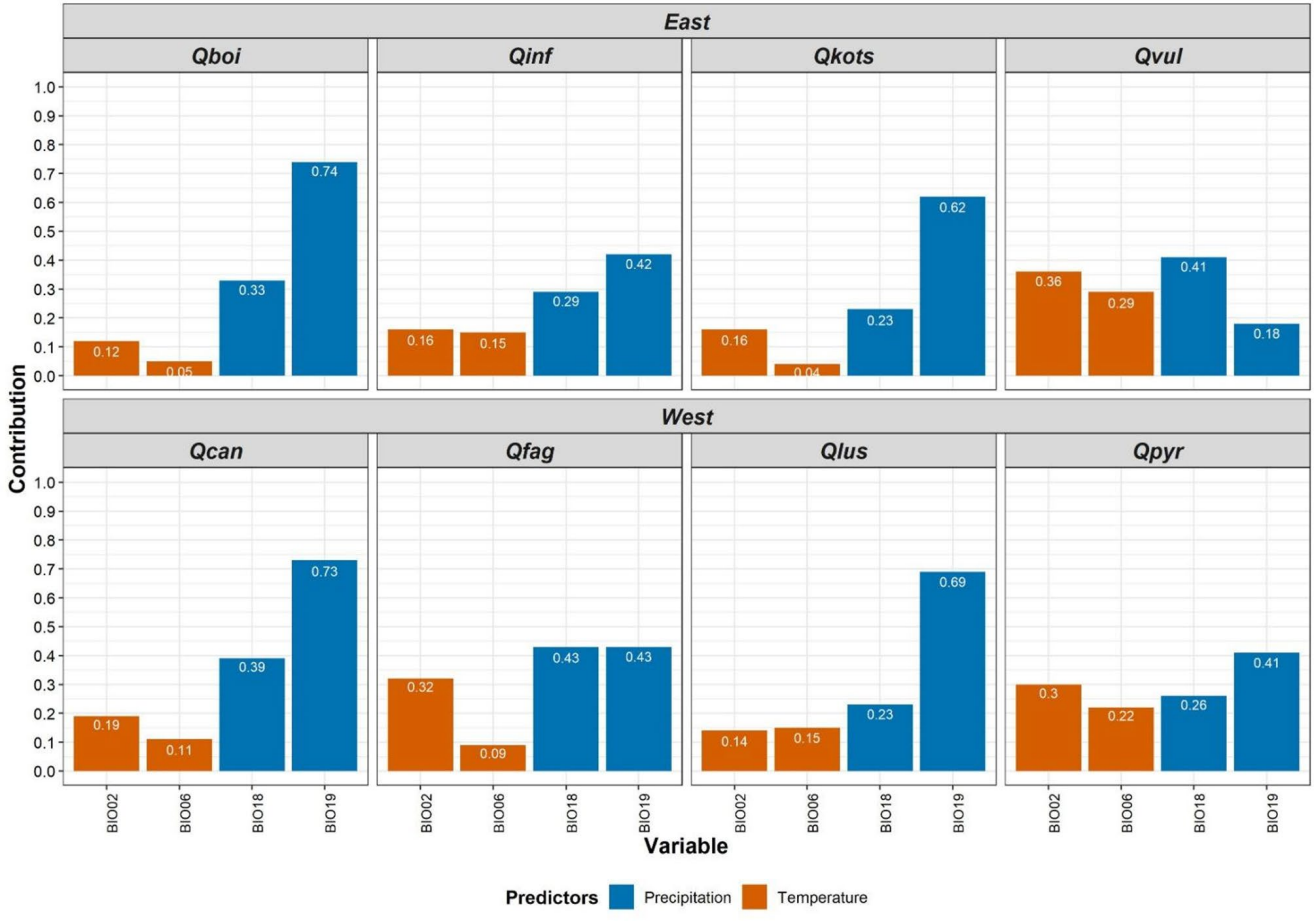
Mean permutation-based variable importance for BIO2, BIO6, BIO18 and BIO19 across the ensemble SDMs for eastern and western species groups. Importance values reflect the decrease in predictive performance after permuting each variable.

Overall, precipitation variables were more important than temperature predictors for most species (Fig. 1). Exceptions were *Q. pyrenaica* and *Q. vulcanica*, where the mean diurnal temperature range (BIO02) was more influential than winter or summer precipitation. Winter precipitation (BIO19) was key for most oaks, except *Q. vulcanica*, which depended more on summer rain (BIO18). Eastern and western taxa showed similar variable patterns, especially *Q. boissieri* and *Q. canariensis*, suggesting biogeographic vicariance (Fig. 1).

Ensemble response curves revealed distinct and non-linear relationships between climatic suitability and the four predictors retained after multicollinearity filtering (Supplementary Fig. S1). Winter precipitation (BIO19) consistently showed positive relationships across species, with suitability increasing rapidly toward intermediate values and stabilizing thereafter, indicating its role in sustaining baseline climatic suitability. In contrast, precipitation of the warmest quarter (BIO18) exhibited predominantly unimodal responses, with sharp declines under low summer rainfall, highlighting sensitivity to increasing summer drought. Temperature-related predictors revealed contrasting roles: minimum temperature of the coldest month (BIO06) showed threshold-like responses, with abrupt suitability losses below species-specific winter temperature limits, while mean diurnal temperature range (BIO02) displayed weaker and more gradual effects, acting as a secondary stressor associated with increasing climatic continentality. Together, these curves clarify how precipitation seasonality governs suitability gradients, while winter cold imposes hard climatic limits across taxa.

Variability in predictor importance across ensemble algorithms was moderate and did not alter the overall ranking of climatic drivers, with precipitation variables consistently dominating over temperature predictors (Supplementary Fig. S2).

### Past-to-present distribution range shifts

#### Geographic range shifts

Most species showed southward latitudinal shifts. Exceptions were northward movements in *Q. pyrenaica* since the Younger Dryas, (YDS onwards), *Q. boissieri* (EH onwards) and *Q. vulcanica* (LH), though the latter expanded only marginally (Supplementary Fig. S3a; Maps 1, 7, 8—Digital Annex). Longitudinal trends varied more: *Q. canariensis* shifted eastwards throughout, *Q. faginea* from YDS onwards, and *Q. pyrenaica* since MH, alongside a coastal contraction during the YDS. *Q. lusitanica* remained mostly stable, except for a coastal contraction in the LH (Supplementary Fig. S3b; Maps 2, 3, 6, 7—Digital Annex).

#### Species range changes (total area and losses)

**Fig. 2:**
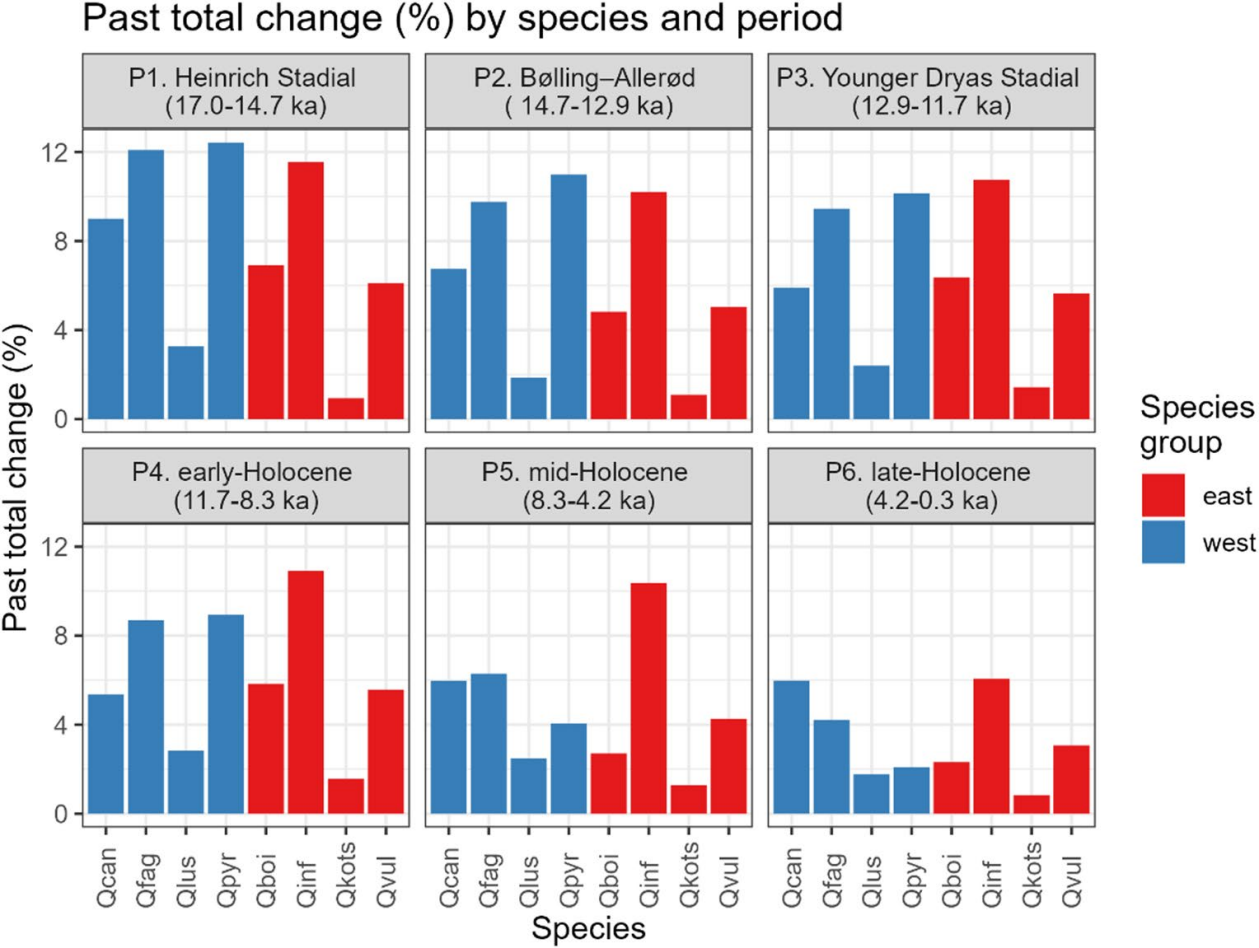
Past-to-present total change (%) by species and period.

Past-to-present projections showed that *Q. faginea* and *Q. pyrenaica*, the most widespread taxa, had the largest range changes up to the Early Holocene (EH). *Q. infectoria* showed the greatest shift during the Younger Dryas (YDS) and maintained marked suitable-area changes throughout the Holocene. In the Mid- and Late Holocene (respectively, MH and LH), *Q. canariensis* showed pronounced changes, along with *Q. infectoria* and *Q. vulcanica*. In contrast, *Q. lusitanica* and *Q. kotschyana* had minor changes throughout (Fig. 2; Supplementary Maps 2–5, 7, 8 — Digital Annex). Suitable-area loss estimates confirmed that *Q. infectoria* had the largest area decrease in most periods, except in the LH, when *Q. canariensis* showed the greatest loss. Narrow-range species (*Q. kotschyana*, *Q. vulcanica*, *Q. lusitanica*) showed minimal losses (Supplementary Fig. S4; Supplementary Maps 2, 4–6, 8 — Digital Annex).

### Present-to-future distribution range shifts

#### Geographic range shifts

**Fig. 3:**
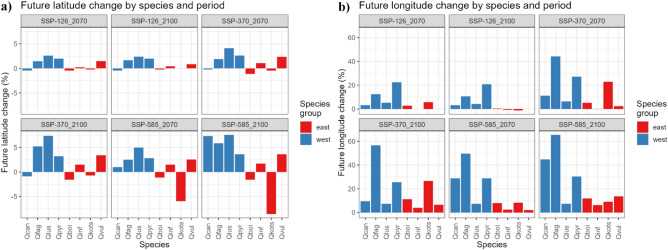
(**a**) Present-to-future latitudinal change by species and period; (**b**) Present-to-future longitudinal change by species and period.

Most circummediterranean oaks are projected to shift northwards, except eastern *Q. boissieri* and *Q. kotschyana*, which may shift southwards under SSP5-8.5 (Supplementary Maps 9, 13—Digital Annex). Major northward shifts are expected for western oaks by 2100 (Fig. 3a; Supplementary Maps 10, 11, 14, 15). Longitudinally, shifts are mostly eastwards, increasing under more pessimistic scenarios, with western species like *Q. faginea*, *Q. pyrenaica* and by 2100 also *Q. canariensis* showing notable changes (Fig. 3b; Supplementary Maps 10, 11, 15—Digital Annex).

#### Species range changes (total area and losses)

**Fig. 4:**
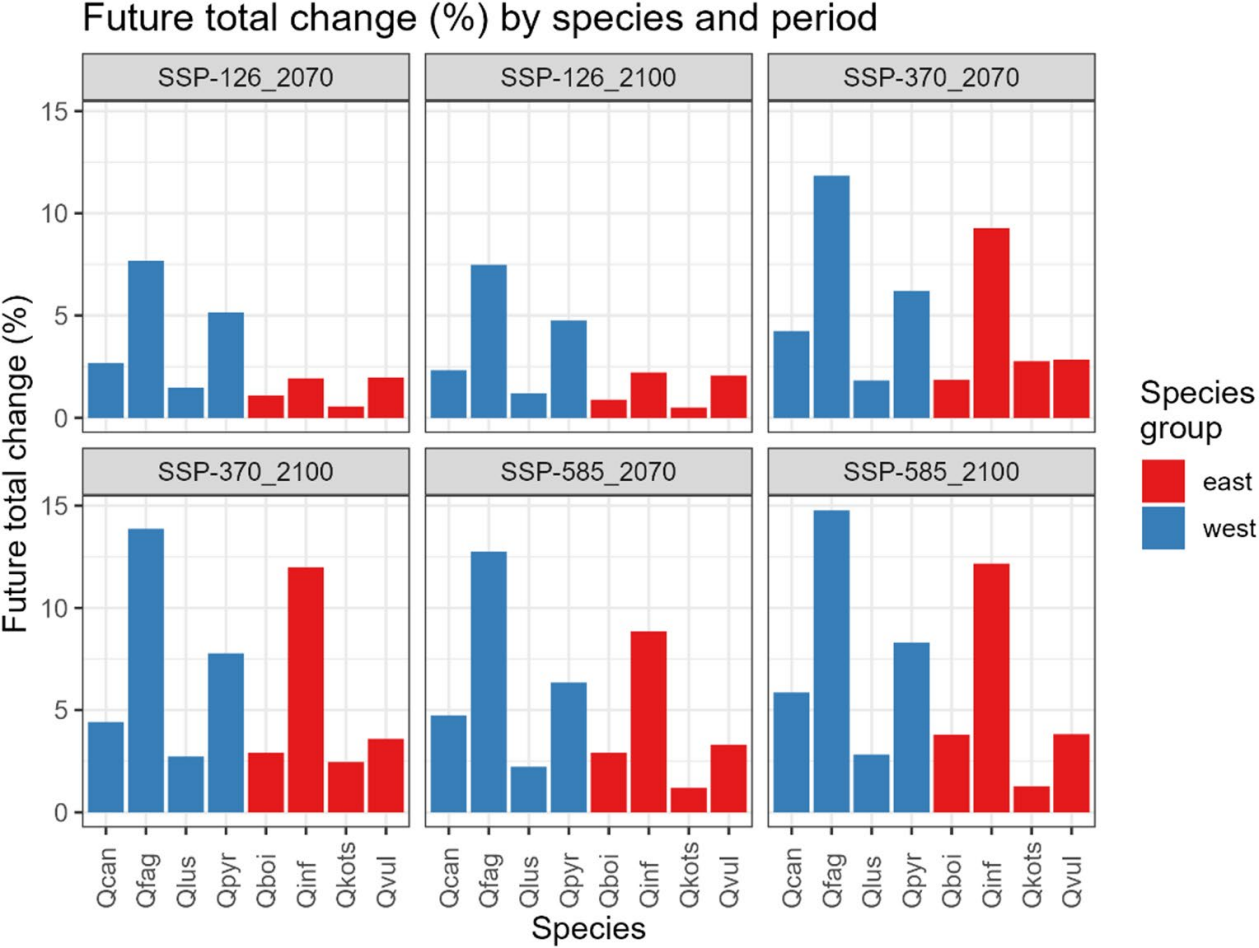
Present-to-future total change (%) by species and period (2070–2100).

Total area change (gains and losses) across six future scenarios (SSPs 126, 370, 585 for 2070 and 2100) shows *Q. faginea* with the largest range shifts, followed by *Q. pyrenaica* under SSP1-2.6. *Q. infectoria* surpasses other species under SSP3-7.0 and SSP5-8.5. Both *Q. canariensis* and *Q. vulcanica* also show significant changes, especially by 2100 (Fig. 4; Supplementary Maps 10–12, 16—Digital Annex).

For total losses, *Q. faginea* remains the species with the greatest decrease, followed by *Q. pyrenaica* under SSP1-2.6. *Q. infectoria* has the second largest losses under SSP3-7.0 and SSP5-8.5, followed by *Q. canariensis* and *Q. vulcanica* (Supplementary Fig. S5; Supplementary Maps 10–12, 16—Digital Annex).

### Future scenarios for vulnerable taxa

**Fig. 5:**
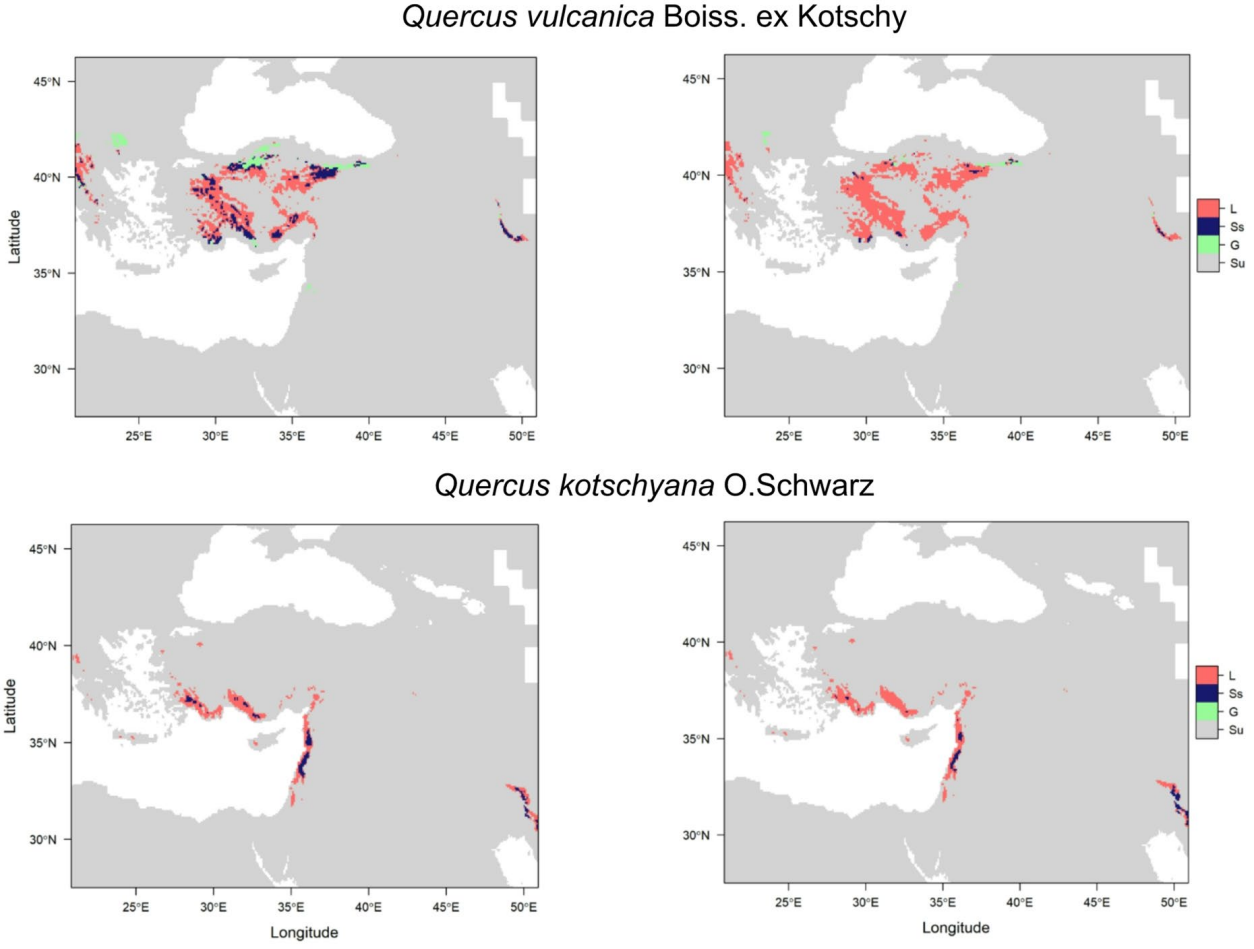
*Q. kotschyana* and *Q. vulcanica* future range shifts (SSP5-8.5) for 2070 (left maps) and 2100 (right maps). L-Loss; Ss-Stable suitable; G-Gain; Su-Stable unsuitable. Species distribution maps were generated by the authors in R v.4.4.1 (https://www.r-project.org/) from ensemble SDM outputs and subsequently assembled and edited in ArcMap v.10.5 (https://www.esri.com/en-us/arcgis).

**Fig. 6:**
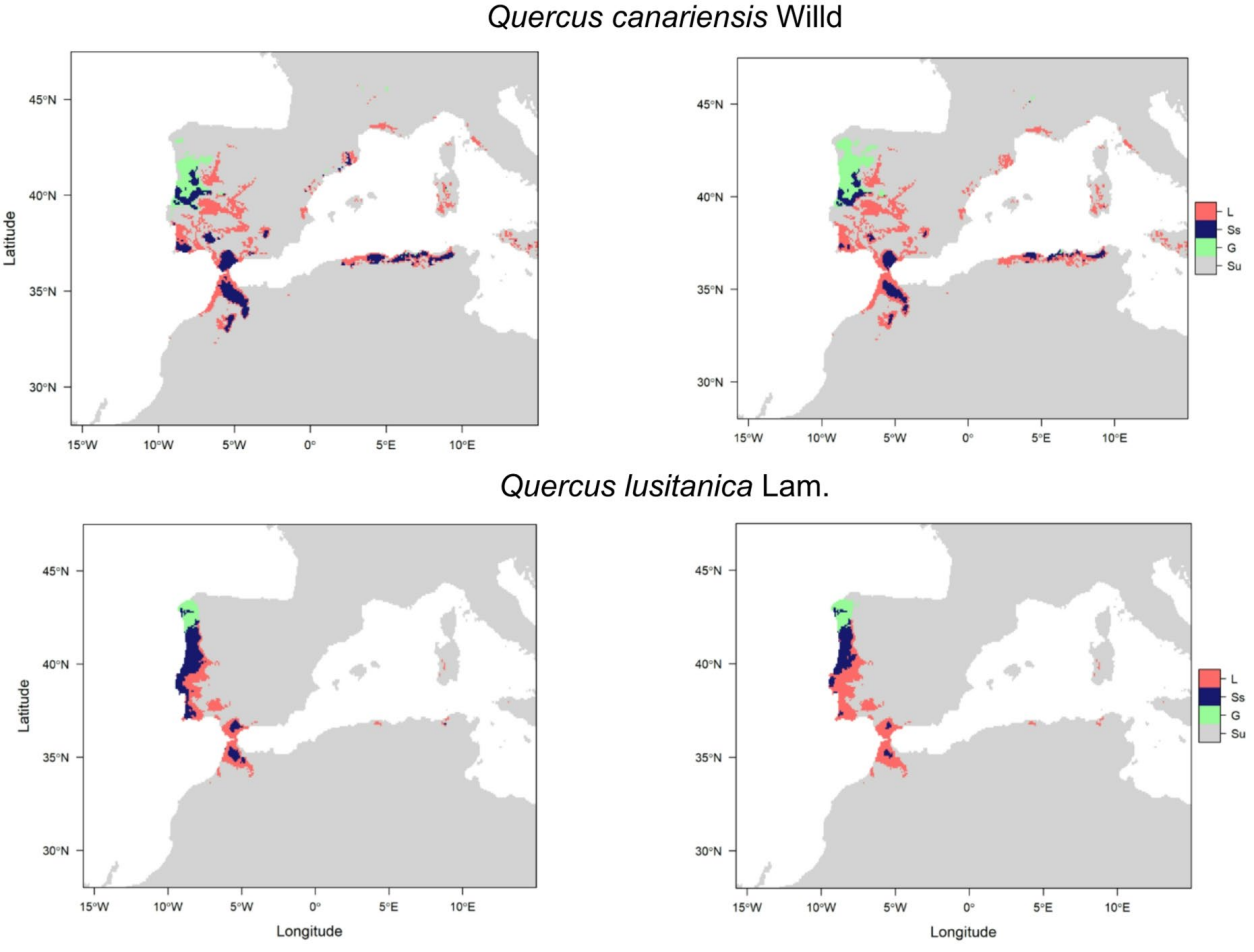
*Quercus lusitanica* and *Q. canariensis* future range shifts (SSP5-8.5) for 2070 (left maps) and 2100 (right maps). L-Loss; Ss-Stable suitable; G-Gain; Su-Stable unsuitable*.* Species distribution maps were generated by the authors in R v.4.4.1 (https://www.r-project.org/) from ensemble SDM outputs and subsequently assembled and edited in ArcMap v.10.5 (https://www.esri.com/en-us/arcgis).

Model projections under the worst-case scenario (SSP5-8.5) for the four narrowly distributed taxa (*Quercus canariensis, Q. lusitanica, Q. kotschyana* and *Q. vulcanica*) reveal major losses in climatically suitable area and extensive range shifts by the end of the century (Figs. 5 and 6).

#### Eastern Mediterranean sensitive taxa

The narrowly distributed *Q. vulcanica* is projected to undergo a general contraction by 2070, retreating towards higher elevations. The easternmost subpopulation (Osmaniye, Turkey) is expected to lose climatic suitability, while others may persist more stably at altitude (Fig. 5). Potential expansion is projected around the northernmost populations (Küre and Ilgaz Mountains, Turkey), suggesting that these areas may offer climatic refuge. By 2100, most subpopulations are predicted to suffer considerable loss, especially in the southeast, including total unsuitability at its type locality in Karadağ (Karaman, Turkey)^1^. Mountainous regions in the western portion of the distribution, notably around Isparta and Ankara, which host well-preserved stands, are likely to be critical for the species’ persistence.

Similarly, the Lebanese endemic *Q. kotschyana*, restricted to Mount Lebanon between 1600 and 2000 m, is highly vulnerable due to its narrow altitudinal range. Projections for 2070 and 2100 indicate no expansion of suitable area and significant losses, potentially restricting the species to the western slopes or pushing it above 2000 m elevation. The overlap between the climatic space retained for *Q. kotschyana* and areas projected as future refugia for *Q. vulcanica* suggests niche convergence between these morphologically and biogeographically related eastern Mediterranean oaks.

#### Western Mediterranean sensitive taxa

*Q. canariensis* is projected to undergo progressive range contraction, with the remaining suitable areas concentrated in Monchique (Portugal), Aracena and Algeciras (Spain), the Rif and Middle Atlas (Morocco), the Algerian Atlas, and Tunisia by 2070. The Catalonian subpopulation appears especially at risk of extinction (Fig. 6). Toward 2100, projections indicate further losses, including total unsuitability in Catalonia and pronounced contraction in the other strongholds, particularly in Portugal and North Africa. Additionally, suitable climate may shift northwards into the northern Iberian Peninsula, where the species is not currently native.

For *Q. lusitanica*, inland subpopulations are projected to decline by 2070, whereas coastal populations may persist. The Aracena Mountains subpopulation may already lose climatic suitability by mid-century. By 2100, the SSP5-8.5 scenario predicts worsening conditions for southern populations, maintaining suitability only in Monchique (Portugal), Algeciras (Spain), and North Morocco. Conversely, centre-western and northern Portugal and Galicia may become key areas of persistence, emerging as future refugia for the species (Fig. 6).

### Associations between past and future range shifts

#### Associations between past-to-present and present-to-future geographic shifts

Associations between past-to-present and present-to-future latitudinal shifts across all species (Supp. Fig. S6a) and species groups (Supp. Fig. S6b) are generally weak. Only eastern Mediterranean species showed a positive correlation, with the most recent period (LH) modestly predicting future shifts (Supp. Fig. S7a). Other periods showed lower and inconsistent correlations. Eastern species exhibited higher latitudinal shift correlation than western ones. When comparing future scenarios with past periods (Supp. Fig. S7b), eastern species had higher *R*^*2*^ under SSP3-7.0/2070–2100 (*R*^*2*^ = 0.12), while western species showed a stronger correlation with SSP5-8.5/2100 (*R*^*2*^ = 0.36).

Correlations between past and future longitudinal shifts (Supp. Fig. S8a, S8b) were also weak overall. However, positive and consistent trends between eastern and western species were found for MH (8.3–4.2 ka; *R*^*2*^ = 0.14–0.16) and LH (4.2–0.3 ka; *R*^*2*^ = 0.22–0.27) (Supp. Fig. S9a). Comparisons with future scenarios (Supp. Fig. S9a,b) revealed lower correlations and inconsistencies across groups. In general, associations between past and future shift trajectories declined from SSP1-2.6 to SSP5-8.5.

#### Associations between past and future range shifts for total area change and loss

**Fig. 7:**
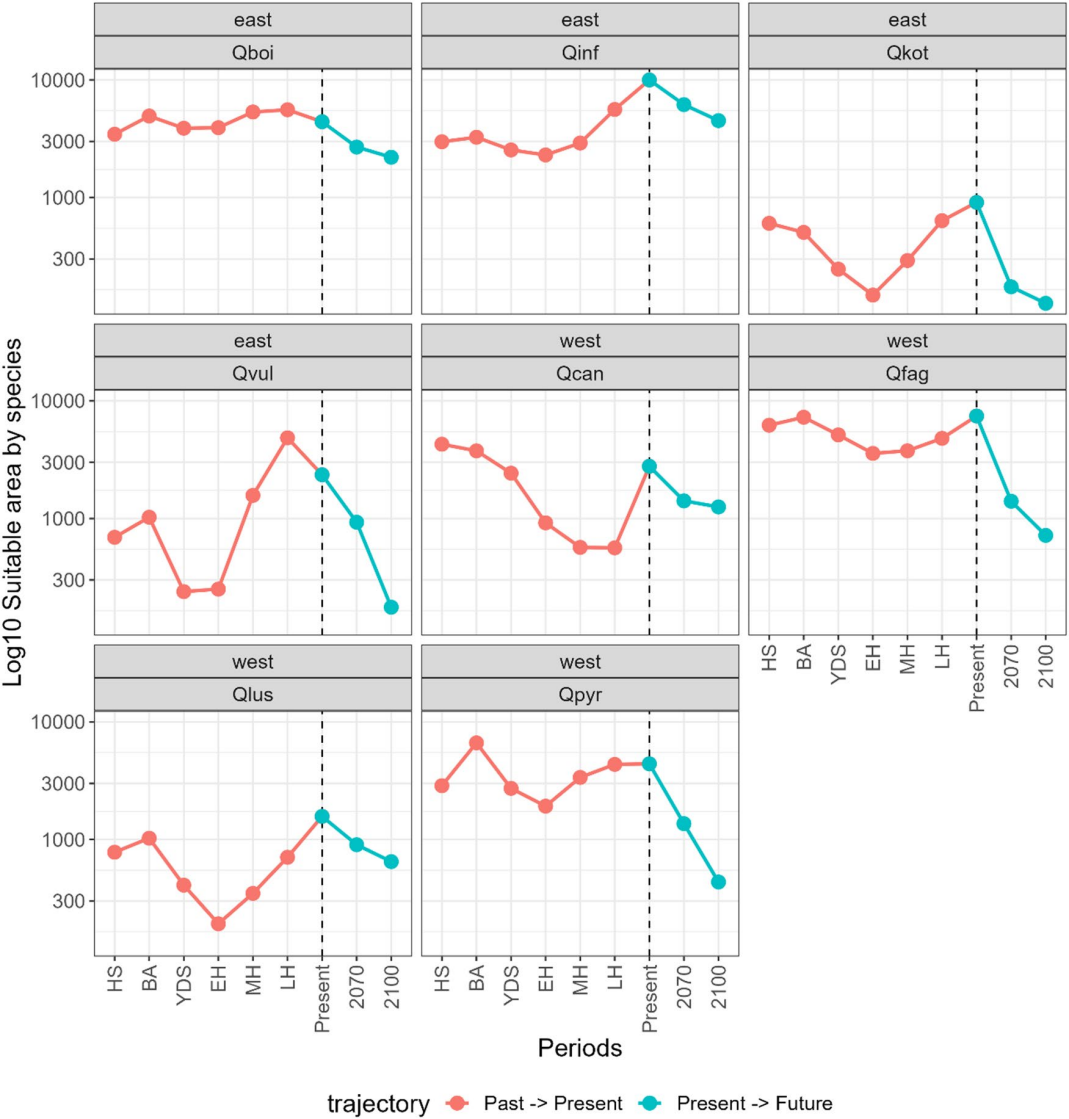
Temporal changes in suitable area per species (Log10 transformed) across all periods (past: HS – Heinrich Stadial; BA—Bølling–Allerød; YDS – Younger Dryas; EH – Early-Holocene; MH – Mid-Holocene; LH – Late-Holocene; Present; and future projection horizons: 2070/SSP5-8.5 and 2100/SSP5-8.5).

Temporal trends in suitable area (Fig. 7) showed associations with climatic periods, except for *Q. canariensis*, which declined from HS to LH. All species expanded towards BA, then declined from YDS to EH. Recovery occurred during MH, stabilizing in LH—except for increases among eastern species (*Q. vulcanica*, *Q. kotschyana*, *Q. infectoria*) and *Q. lusitanica* in western Iberia. Future projections show smaller losses for *Q. boissieri*, *Q. infectoria*, *Q. canariensis*, and *Q. lusitanica*; greater losses are projected for *Q. vulcanica*, *Q. pyrenaica*, and *Q. faginea* (Fig. 7).

**Fig. 8:**
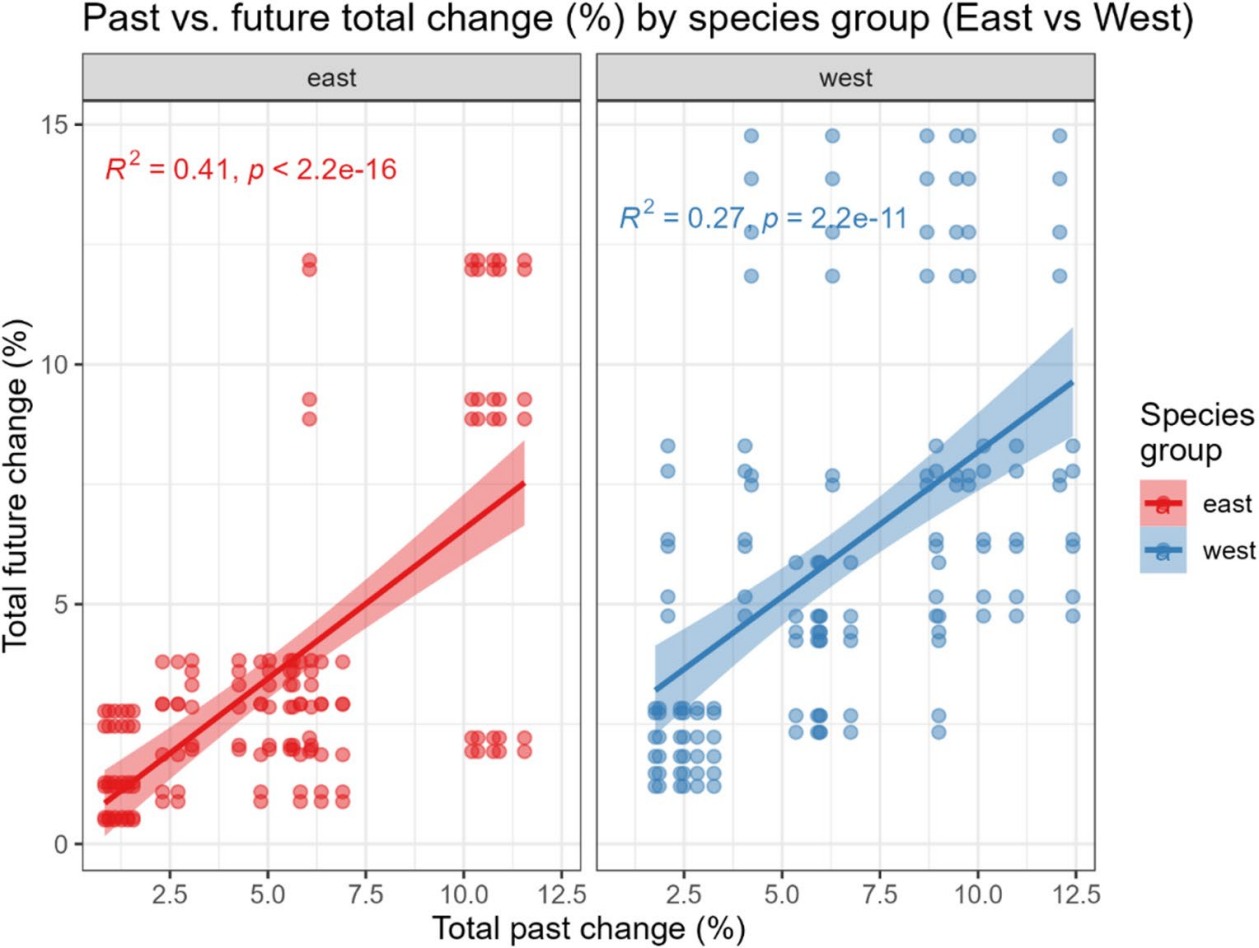
Past-to-present vs present-to-future total change (%) by species group (East vs West). The plot shows all, i.e., many-to-many, associations between past-to-present and present-to-future range shifts for all species within each regional east or west species subgroup.

Overall, correlations between past-to-present and present-to-future range shifts are stronger for area-related metrics (e.g., total change/loss) than for geographic shifts. Total distribution change is positively correlated across all periods (Supp. Fig. S10; *R*^*2*^ = 0.35, *p* < 0.001), especially when split by group: eastern species (*R*^*2*^ = 0.41, *p* < 0.001) vs western (*R*^*2*^ = 0.27, *p* < 0.001) (Fig. 8).

**Fig. 9:**
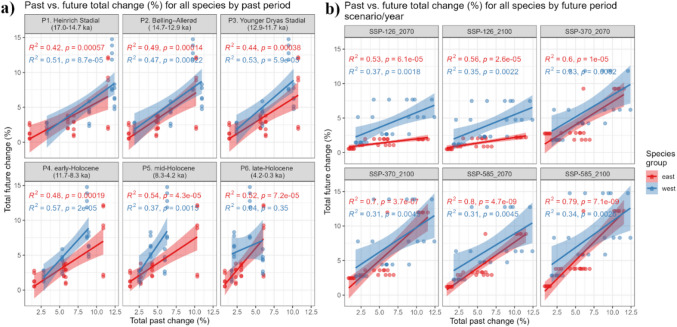
Past-to-present vs present-to-future total change (%) by climatic period and future scenario. (**a**) Associations grouped by past period; (**b**) associations grouped by future scenario/year. Past periods include phases of climatic deterioration, Heinrich Stadial 1 (HS; 17.0–14.7 ka) and Younger Dryas Stadial (YDS; 12.9–11.7 ka), and phases of climatic amelioration, Bølling–Allerød (BA; 14.7–12.9 ka), Early Holocene (EH; 11.7–8.3 ka), Mid Holocene (MH; 8.3–4.2 ka) and Late Holocene (LH; 4.2–0.3 ka). The plot shows one-to-many associations between past-to-present and present-to-future range shifts within each regional subgroup. In panel (**a**), for example, the top-left plot represents species’ range shifts from Heinrich Stadial 1 to the present on the x-axis versus all present-to-future horizons on the y-axis. Red and blue points, regression lines and statistics represent eastern and western Mediterranean species groups, respectively.

Comparisons between each past period and all future horizons (Fig. 9a) show higher positive correlations for western Mediterranean species, except during the BA and recent Holocene (MH, LH). Thus, eastern oaks’ past shifts correlate more strongly with future projections over the last six millennia. Conversely, eastern oaks’ future shifts correlate more strongly with past changes (Fig. 9b), especially under extreme scenarios: SSP3-7.0 (*R*^*2*^ = 0.60–0.70, *p* < 0.001) and SSP5-8.5 (*R*^*2*^ = 0.79–0.80, *p* < 0.001). Western species show lower and more consistent values across scenarios (*R*^*2*^ = 0.31–0.37, *p* < 0.01).

Percentage losses between past and future periods mirrored total area change results, with positive correlations for all species and combinations (Supp. Fig. S11a). Eastern oaks showed higher *R*^*2*^ values (0.36) than western ones (0.22) (Supp. Fig. S11b). When analyzing correlations for suitable area losses per past period (Supp. Fig. S12a), eastern species had stronger associations for BA, LH, and MH (*R*^*2*^ = 0.48–0.54, *p* < 0.001), while western species correlated more with HS, YDS, and EH (*R*^*2*^ = 0.51–0.57, *p* < 0.001).

Total percentage loss correlations remained stable across scenarios for western species (R^2^ = 0.26–0.27), whereas eastern oaks showed strong correlation under SSP5-8.5 (R^2^ = 0.76; Supp. Fig. S12b).

## Discussion

Our results reveal moderate to strong correlations between past and future range dynamics since Heinrich Stadial 1 (~ 17 ka), with species experiencing greater past shifts also facing more severe future range contractions, especially under pessimistic scenarios. Similar trends in eastern and western taxa underscore a biogeographic divide in Mediterranean oaks ^38^, with eastern species facing harsher Holocene conditions than their western counterparts. Although hindcasts provide valuable ecological insight, projected future climates may exceed Late-Quaternary climatic envelopes, limiting the temporal transferability of SDMs^8,39^. Quantitative validation of species-level hindcasts is constrained because Quercus pollen cannot be reliably identified to species level^40^; paleorecords nonetheless provide useful context for broad trends such as Holocene aridification and east–west contrasts^22–24^. Accordingly, concordance between past and future responses is interpreted as ecological guidance rather than strict predictive equivalence. Regression summaries are therefore treated as exploratory descriptors rather than causal tests^35,40^.

### Past and future range shifts

Precipitation was the main driver of past and future range shifts, followed by temperature seasonality, particularly for *Q. vulcanica* and *Q. pyrenaica*, as suggested by Stephan et al.^41^ and Pérez-Luque et al.^42^. Correlations between past and future projections varied by species and scenario, with stronger links under SSP5-8.5 (Figs. 7, 8), consistent with projected rainfall declines. Eastern species showed stronger correlations during climatic amelioration (BA, MH, LH), while western taxa aligned with deteriorative periods (HS, YDS), indicating a longitudinal asymmetry supported by palynological evidence^23^. This reinforces the asymmetric association between past dynamics and future range shifts, consistent with more contrasting oscillations in the western basin.

The moderate variability observed among ensemble algorithms does not affect the dominance of precipitation-driven constraints, supporting the robustness of the inferred climatic controls on past and future range dynamics.

The species-specific response curves align closely with the known ecology and biogeographic affinities of submediterranean marcescent oaks, strengthening the ecological interpretation of both past and future projections. *Quercus canariensis*, a mesophilous oak associated with Atlantic climates, shows strong dependence on high winter precipitation and limited tolerance to summer drought, consistent with its occurrence in humid valleys and mountainous areas characterised by low precipitation seasonality and frequent fog formation. In contrast, *Quercus faginea* exhibits broad tolerance to winter cold, summer drought and precipitation seasonality, reflecting its marked ecological plasticity and persistence across continental, montane and submediterranean environments^4,11^.

*Quercus pyrenaica* displays high tolerance to winter cold and moderate resistance to summer drought, supporting its tendency to track colder conditions upslope and maintain wide elevational ranges^31,43^, whereas *Quercus lusitanica* emerges as strongly cold-intolerant and tightly linked to rainy winters, confirming its strict Atlantic affinity and dependence on temperate, humid climates^4^. Eastern Mediterranean taxa mirror these patterns through vicariant relationships: *Quercus boissieri* shows climatic responses comparable to *Q. canariensis*, while *Quercus kotschyana* and *Quercus vulcanica* closely resemble the cold-tolerant, montane niche of *Q. pyrenaica* (see Fig. 1 and Supplementary Fig. S1).

By including eastern oaks phylogenetically related to western taxa, we complement previous studies on marcescent forests^4,11^ and revamp their biogeographic vicariance^44^. This is evident in shared predictor profiles between *Q. boissieri* and *Q. canariensis*, and between *Q. vulcanica* and *Q. pyrenaica* (Fig. 1). Our results support the subdivision of European white oaks^16^ into two taxonomic groups defined by Schwarz^17^ (*Dascia* vs *Galliferae*) (See Supplementary Data S1). Eastern Dascia species (*Q. kotschyana*, *Q. vulcanica*) show overlapping projected ranges (Supplementary Maps 5, 8, 13, 16 — Digital Annex), suggesting that niche stability and past vicariance may have shaped their speciation and retention of ancestral traits^45,46^.

Past geographic shifts broadly mirror the patterns described in the Results, with marked displacement in some taxa across Late-Quaternary transitions (Fig. 2; Supplementary Maps 1–8—Digital Annex). These historical dynamics provide context for the projected future redistributions under increasing climatic instability (Supplementary Fig. S4).

Future scenarios project significant northward shifts in climatic suitability, often beyond current ranges (Figs. 2, 3, 6; Supplementary Fig. S5; Supplementary Maps 9–16 — Digital Annex). Among taxa, projected displacement is greatest in the most widespread species (Figs. 3, 6; Supplementary Fig. S5; Supplementary Maps 9–16 — Digital Annex). These shifts may shrink the submediterranean ecotone, favouring replacement by dry-adapted evergreen oaks despite northward expansions^4,47^. Conversely, marcescent oaks could substitute deciduous oaks at the southern limits of temperate forests (Figs. 2, 3; Supplementary Fig. S5; Supplementary Maps 9–16 — Digital Annex).

Past trajectories in suitable area confirm critical phases of successive losses for all species from the Bølling-Allerød to the Early Holocene (Fig. 7). These patterns differ between broadly distributed species (e.g., *Q. faginea*, *Q. pyrenaica*) and narrowly distributed oaks (*Q. kotschyana*, *Q. canariensis*, *Q. vulcanica*), despite similar trends (Fig. 7).

### East–West Biogeographic Dynamics and Vicariant Patterns

Correlations between past and future range shifts highlight an East–West asymmetry, with eastern Mediterranean oaks showing stronger links between Holocene fluctuations and future scenarios (Figs. 6, 8, 9; Supplementary Figs. S6–S12; Supplementary Maps 1–16 — Digital Annex). This agrees with palynological records indicating wetter and cooler summers in the western basin that favoured Iberian white oak persistence^22,23,28^, contrasting with pronounced summer aridity in the Near East^24^.

This pattern reflects the classic amphi-Mediterranean biogeographic model, with lineages surviving at both extremes of the basin but scarce in the centre^1,5^.

The climatic overlap of *Q. boissieri* and *Q. canariensis* illustrates this (Fig. 1; Supplementary Maps 5, 8, 13, 16 — Digital Annex), rooted in the fragmentation of the Tertiary Laurel Forest biome and maintained by persistence in glacial refugia^27^. Comparable East–West splits are found in *Prunus lusitanica* and *Ilex aquifolium*^48,49^, lineages typical of mild mesophytic habitats overlapping areas of high suitability for these oaks^50^. The niche overlap of *Q. kotschyana* and *Q. vulcanica* with *Q. frainetto* supports relictual niche conservatism, consistent with Neogene climatic disruption as a driver of the present East–West oak disjunction^51^.

### Future conservation perspectives of sensitive taxa and submediterranean oak forests

In both subregions, sensitive oaks show consistent contraction patterns toward high-altitude refugia or latitudinal shifts, mainly due to precipitation changes. This effect is especially evident for eastern species such as *Q. kotschyana* and *Q. vulcanica*, projected to suffer significant losses in climatic suitability by the end of the century (Figs. 2, 3, 4, 6; Supplementary Fig. S5). For *Q. kotschyana*, projected upslope movement in Mount Lebanon may be constrained by degraded soils, lower minimum temperatures, and increased frost-wind damage. *Q. vulcanica* shows a similar trend, with southeastern populations declining and climatic suitability shifting to northeastern Anatolia—particularly the southern Kaçkar foothills, where conditions are more continental than in the humid coastal zone (Figs. 2, 3, 4, 6; Supplementary Fig. S5).

In the western Mediterranean, *Q. canariensis* appears most vulnerable. Worst-case projections (SSP5-8.5) predict significant declines in SW Iberia and N Africa, mainly from decreasing annual precipitation, with possible disappearance of the Catalonian and Aracena subpopulations by 2100 (Figs. 4, 5, 7, 8; Supplementary Fig. S5). *Q. lusitanica* is projected to shift northward, with SW populations retreating to higher elevations (Monchique, Algeciras), followed by movement into NW Iberia, where reduced winter cold and sufficient rainfall favor persistence. Inland populations like Aracena, São Mamede, and the Tagus-Sado Basin, will likely become unsuitable (Figs. 2, 3, 5, 6; Supplementary Fig. S5).

Projected past and future climatic refugia highlight stable zones critical for long-term persistence of sensitive oaks. These areas should guide conservation and climate adaptation strategies^8,52,53^. Their location in a key phylogeographic hotspot reinforces their value as reservoirs of endemic and genetic diversity^4,54–57^.

Our results emphasize that robust taxonomic filtering and reliable occurrence data are essential for accurate SDM-based conservation. Future efforts should address taxa with unresolved classification^16^ (see Supplementary Data S1) and expand field surveys, expeditions, and herbarium research, which remain fundamental to address the biodiversity crisis. Recent work on *Arbutus* similarly shows that incorporating palaeobotanical occurrences into ecological niche models can refine past suitability estimates, reinforcing the value of integrating modelling with curated botanical, fossil and taxonomic evidence^58^. Such efforts help refine hindcasts and forecasts beyond static LGM or Holocene scenarios^7,27,29^, improving predictions for critical deglaciation intervals. Moreover, moving beyond static time-slices such as the LGM or mid-Holocene by incorporating more continuous paleoclimatic intervals enhances the capacity to reconstruct contrasting climatic trends and evaluate ecological responses with greater realism^5,27^.

Several limitations should be considered when interpreting our results, particularly regarding model transferability under non-analogue climates^8,35^, the assumption of niche stability through time, and the exclusion of dispersal, biotic interactions and non-climatic constraints. Dynamic factors such as dispersal processes, species interactions (e.g., competition or facilitation), and non-climatic constraints (e.g., soil or disturbance regimes) remain unaccounted for, despite their known role in shaping future range responses^32,35^. Integrating paleoecological proxies such as fossil pollen or phylogeographic patterns can further improve temporal reconstructions and inform model validation^7,27,29^. While these additions increase complexity and data requirements, they represent important directions for future model refinement and conservation planning under climate change.

## Conclusions

Using retrospective hindcasts reveals how species responded to abrupt climatic shifts in the past, providing key insights that help anticipate the scale and spatial pattern of their projected range shifts by 2100. While SDMs inform conservation, they rarely integrate past dynamics. Here, we show how evidence of past range shifts refines forecasts for eight circummediterranean white oaks and supports forest preservation. Strong model performance and the role of precipitation seasonality confirm oaks as suitable models for paleobiogeographic studies, revealing historical suitability dynamics and evolutionary-biogeographic patterns in transitional zones across Europe, North Africa and the Near East.

For narrowly distributed taxa, worst-case scenarios project major losses for *Q. canariensis, Q. lusitanica, Q. kotschyana* and *Q. vulcanica*, with northward and coastal shifts. This confirms *Q. kotschyana, Q. vulcanica* and *Q. canariensis* as urgent conservation targets, warranting IUCN reassessment^20^. Small populations and range shifts into unreachable areas demand active conservation planning. We argue that combining past range dynamics with future forecasts should become standard to guide policies that reduce climate-driven biodiversity loss.

### Methods

**Fig. 10:**
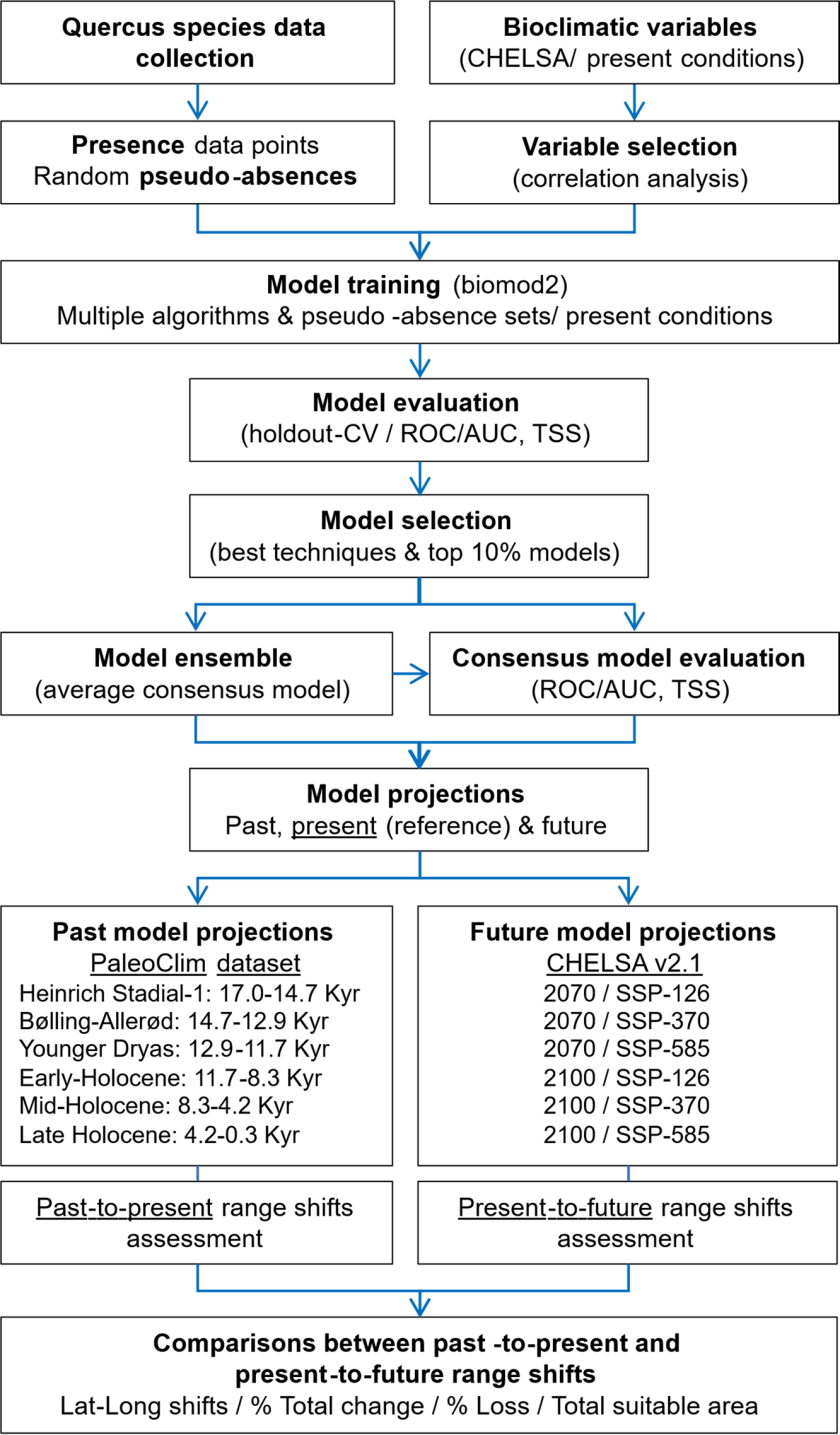
Modelling workflow.

We compiled and harmonized *Quercus* presence records, selecting bioclimatic predictors while minimizing multicollinearity (Fig. 10). Pseudo-absences were generated to complement presence-only data. Models were trained using the biomod2 R package Thuiller, et al.^59^, and partial models were ranked by evaluation. The best-performing models were combined into an ensemble for past, present and future projections. Range shift indices (latitude/longitude shifts, total area change) allowed comparison of species trajectories across timeframes^29^.

### Study area

**Fig. 11:**
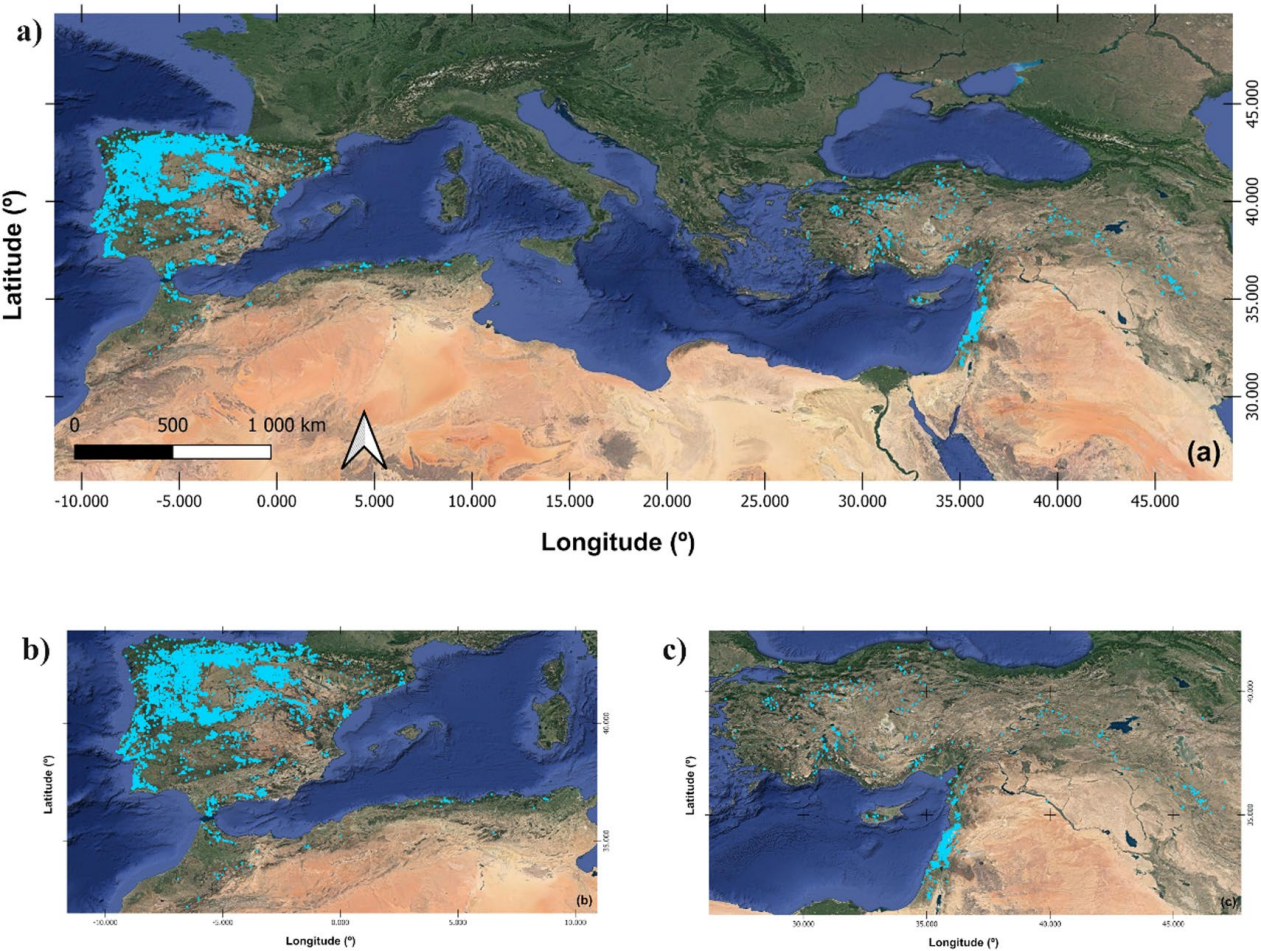
(**a**) Study area and selected bounding box with all oak species locations. The subsequent division into two sub-boxes featuring (**b**) Western distributed oaks species and c) Eastern distributed oaks species. Maps were generated by the authors in R v.4.4.1 (https://www.r-project.org/) from ensemble SDM outputs and subsequently assembled and edited in ArcMap v.10.5 (https://www.esri.com/en-us/arcgis).

The study area covers most of the Mediterranean Basin (8.1 million km^2^). It includes Eurosiberian and Mediterranean regions with varied geology and a typical Mediterranean climate^14^. We split the area into western and eastern sub-basins using a 500 km buffer around species’ distribution limits (Fig. 11).

### Focal species and occurrence data

We focused on eight circummediterranean marcescent oaks traditionally grouped within Section Quercus^16,17^. Full taxonomic details and occurrence data sources, including fieldwork, herbaria and GBIF records, are provided in Supplementary Data S1 and Tables S1, S2. Occurrence data were subjected to rigorous scrutiny following procedures established in our previous work on Mediterranean white oaks^4^, which included specimen-by-specimen revision of major herbaria such as the Paris Herbarium (P, MNHN). This taxonomic curation allowed us to resolve misidentifications, verify historical records and ensure the reliability of GBIF-derived occurrences inherited from these collections. We also provide species-specific occurrence maps compiling field, herbarium and filtered GBIF records (Supplementary Fig. S13), complementing Supplementary Tables S1, S2 and the Digital Annex dataset.

### Environmental variables (predictors)

Models were based on regional bioclimatic predictors relevant to the species’ distribution and niche. For hindcasts, we used 19 variables from six paleoclimate simulations in PaleoClim v1.0 (http://www.paleoclim.org/) (Supplementary Table S3)^60^, covering abrupt climate shifts since ~ 17.4 kyr BP, including the Heinrich Stadial, Younger Dryas, Bølling-Allerød, and the Early, Mid- and Late Holocene.

Forecasts used CHELSA v2.1 (https://chelsa-climate.org/downloads), with the same 19 variables for the 1981–2010 baseline and two future periods (2070, 2100). Climate projections were averaged from five GCMs (GFDL-ESM4, IPSL-CM6A-LR, MPI-ESM1-2-HR, MRI-ESM2-0, UKESM1-0-LL) under CMIP6, across three SSP scenarios (SSP1-2.6, SSP3-7.0, SSP5-8.5), selected for strong performance in Europe^61^. We used the CMIP6 multi-model mean to emphasise the robust consensus signal across GCMs, following common practice for ecological projections and to minimise the influence of model-specific anomalies. All data were reprojected to a 10 × 10 km grid (WGS1984 UTM30N). PaleoClim paleoclimate layers represent 30-year climatic windows reconstructed from GCM anomalies downscaled onto high-resolution baselines, providing bioclimatic surfaces for each Late-Quaternary interval. CHELSA future projections similarly represent 30-year averages centred on 2070 and 2100. For each period, we used the CMIP6 multi-model mean across five GCMs to emphasise the shared consensus signal while reducing the influence of model-specific anomalies. After collinearity filtering (r < 0.7), we retained four variables: BIO2 (diurnal temperature range), BIO6 (minimum temperature of coldest month), BIO18 (precipitation of warmest quarter), and BIO19 (precipitation of coldest quarter), allowing analysis of seasonal effects on white oaks.

To facilitate ecological interpretation of predictor effects, ensemble response curves were generated for all climatic variables retained after multicollinearity filtering; methodological details and graphical outputs are provided in the Supplementary Information (Supplementary Fig. S1).

The 19 bioclimatic variables used in both PaleoClim and CHELSA correspond to the standard BIO1–BIO19 set; full definitions are provided in the PaleoClim and CHELSA documentation (Table S3). Average variable importance across species and between eastern and western groups is presented in Fig. 1, allowing direct comparison of the relative influence of each predictor.

### Model calibration and evaluation

Species Distribution Models (SDMs) were developed in R 4.2.0 using the *biomod2* package^59^, which applies an ensemble approach combining statistical and machine learning algorithms. Nine algorithms were used (GLM, GBM, GAM, CTA, ANN, FDA, MARS, RF, MAXENT), with default parameters, except for GAM (smoothing term k = 4) and GBM (n.trees = 2000) to reduce overfitting^62^. Due to the presence-only nature of data, ten pseudoabsence (PA) sets were generated, matching the number of presences to balance prevalence and ensuring minimum distances of 10 km without overlap^63,64^. Prevalence was set at *p* = 0.5 (default). Models were trained under current conditions, and projections were obtained by replacing climate layers with PaleoClim and CHELSA v2.1 datasets. Eastern and western species were modeled using bounding boxes defined by occurrence subsets plus a 500 km buffer. Models were evaluated using 20-fold holdout cross-validation (80/20 split), and performance was assessed with AUC, TSS, Sensitivity, and Specificity^59^. AUC was classified from poor (0.5–0.6) to excellent (> 0.9); TSS from very poor (≤ 0.2) to excellent (> 0.8) ^65^. TSS can assume values between − 1 and 1 and can be classified as excellent (TSS > 0.8), good (0.6–0.8), fair (0.4–0.6), poor (0.4–0.2) and, very poor (TSS ≤ 0.2)^66^. The TSS-maximizing threshold was used to convert continuous predictions into binary presence/absence maps^67^. We adopted an ensemble modelling framework because individual algorithms differ in their sensitivity to data structure and response shapes, and ensembles reduce algorithm-specific biases, improving robustness across past and future projections. Each algorithm was evaluated through repeated cross-validation, and only models with TSS > 0.7 contributed to the final ensemble. Ensemble predictions were computed as weighted means, with weights proportional to each algorithm’s TSS score. Pseudo-absences were generated using biomod2’s random-sampling procedure within the accessible background, excluding a buffer around known presences to reduce spatial autocorrelation. For each species, 10,000 pseudo-absences were used, following standard ensemble modelling practice. Given the high-quality and taxonomically curated nature of the presence dataset, no additional spatial thinning was applied. Multicollinearity among predictors was evaluated using pairwise Pearson correlations, retaining only variables with r < 0.7 (BIO2, BIO6, BIO18 and BIO19). Variable importance was computed using biomod2’s permutation procedure, which quantifies the reduction in model performance when a predictor is randomly permuted while others remain constant. Mean and standard deviation of variable importance were calculated across ensemble algorithms to quantify variability among modelling approaches. To ensure transparency and reproducibility, our modelling workflow and reporting follow the recommendations of the Zurell et al.^68^ SDM reporting protocol, including explicit documentation of occurrence curation, predictor selection, collinearity filtering, pseudo-absence design, algorithm evaluation, ensemble construction and projection procedures.

### Comparisons between past-to-present and present-to-future range shifts

We overlapped model projections to quantify range shifts, calculating the proportion of stable areas (suitable or unsuitable), losses, and gains. Analyses were run in R using the raster package^69^. Current conditions were used as the baseline for all comparisons. As indices of change, we used total change (T_c; Eq. 1), loss (Eq. 2), and latitudinal/longitudinal shifts in species distribution centroids (Eqns. 3, 4). A many-to-many matrix was built combining all past-to-present and present-to-future comparisons per species, enabling comprehensive statistical screening.

1 $$\% Tc = \left( {\frac{L + G}{{S + G + L}}} \right) \times 100\,{\mathrm{with}}\,S = S_{u} + S_{s}$$

2 $$\%L=\left(\frac{L}{S+G+L}\right)\times 100.$$

${p}_{i}=({\varphi}_{i},{\lambda}_{i})$, with i = {1,…,n}, equal to the longitude (*φ*) and latitude (*λ*) of all suitable locations (*n*, as pixels).

**Table:** 

Current projection (baseline)	Past or future model projections	
	Unsuitable	Suitable
Unsuitable	Stable unsuitable (${S}_{u}$)	Gain (*G*)
Suitable	Loss (*L*)	Stable suitable (${S}_{s}$)

$c(\varphi ,\lambda )=(median\left(\left\{{\varphi}_{i}\right\}\right), median(\{{\lambda}_{i}\}))$, with as the median centroid (*c*) of each species distribution. The percent relative shift in longitude and latitude are given for past and future range shifts as:

3 $$\% c(\varphi ,\lambda ) = \left( {\frac{{c(\varphi ,\lambda )_{past} - c(\varphi ,\lambda )_{cur} }}{{c(\varphi ,\lambda )_{cur} }}} \right) \times 100$$

4 $$\% c(\varphi ,\lambda ) = \left( {\frac{{c(\varphi ,\lambda )_{fut} - c(\varphi ,\lambda )_{cur} }}{{c(\varphi ,\lambda )_{cur} }}} \right) \times 100$$

Comparisons between past-to-present and present-to-future range shifts were summarised using simple linear regressions, with R^2^ quantifying their association magnitude. These regressions serve as exploratory descriptors of general tendencies rather than inferential models, as they are not intended to test causality or assume independence among time periods or species. Their purpose is to provide a heuristic comparison of the magnitude and direction of shifts across temporal scales. Panel regressions examined: (i) differences between eastern and western species; (ii) how each past period aligns with all future scenarios (by year and SSP); and (iii) the reverse—how each future scenario aligns with all past periods. These comparisons help identify species groups or time intervals with stronger continuity in range dynamics. All analyses were conducted in R version 4.2.

## Supplementary Information

Below is the link to the electronic supplementary material.

Supplementary Material 1

## Data Availability

Data information and code for analysis and model development, will be made available and released upon acceptance for publication in an open repository (e.g., Zenodo)
