# Supplementary material for: Past projections of submediterranean oaks unveil future range shifts of vulnerable taxa under climate change
Source: Sci Rep. 2026 Jun 4;16:25611. doi: 10.1038/s41598-026-56522-5 (PMC13478631; doi:10.1038/s41598-026-56522-5)
Supplement: Supplementary file 1 [file 41598_2026_56522_MOESM1_ESM.docx]

**10. Supplementary Information**

**Supplementary Data S1** - Focal taxa and occurrence data

The present study benefited from previous taxonomic research, exclusively directed to the study of the type materials of most of the circummediterranean oaks, deposited at the Paris Muséum National d' Histoire Naturelle. This revealed to be crucial for the presented nomenclatural and taxonomic endorsements and was done through a SYNTHESIS Grant (FR-TAF-280). This analysis was further complemented with the available online collections of the Smith Herbarium of Linnean Society (LINN-HS!), the herbarium of the Botanic Garden and Botanical Museum of Berlin-Dahlem (B-W!), the Herbier de l’Université de Montpellier (MPU), the Webb Herbarium from Herbarium Universitatis Florentinae (FI-Webb), the Herbarium from the Conservatoire et Jardin botaniques de la Ville de Genève (G), the Herbarium from Instituto Nacional de Investigación y Tecnología Agraria y Alimentaria (MAIA), the Herbarium from the Swedish Museum of Natural History (S) and the Herbarium from the Royal Botanic Gardens (K). Furthermore, we revised the Iberian oak collections of more fourteen Herbaria, spanning ten Portuguese (AVE, BRESA, COI, HVR, LISE, LISFA, LISI, LISU, PO and UEVH); three Spanish (BCN; HSS and MA) and one from the Netherlands (L) ^1^.

We sustain the focal taxa, based in the original proposals of Lamarck ^2^, Willdenow ^3,^Willdenow ^4,^Olivier ^5^, Boissier ^6^, Kotschy ^7^ and Schwarz ^8^, from which taxonomic circumscription and nomenclatural endorsements were revisited by Avishai ^9^, Stephan and Teeny ^10^, Öney, et al. ^11^ and Yücedağ, et al. ^12^ for the near eastern oaks, while for the Iberian Peninsula and north Africa, other authors like Vasconcellos and Franco ^13^, and more recently Vázquez, et al. ^14^ and Vazquez, et al. ^15^, as follows:

**Section *Quercus*** Denk, et al. ^16^

**Subsection *Galliferae*** ^17^ Gürke ^18^

***Quercus canariensis* Willd.** (1809: 975)

The Algerian oak is a relictual tree, with five small subpopulations in the Iberian Peninsula ^19-21^. In Europe it occurs above siliceous bedrock, always in mesophytic conditions, in spite of being largely distributed above limestones in Africa. It has an higher demand on annual and summer precipitations, when compared with the remaining western *Galliferae* species ^19-21^. It is Critically Endangered in Portugal ^22^ and has a Data Deficient status according to Carrero, et al. ^23^. Its conservation status is ambiguous since it is known to form hybrid swarms with other sympatric oaks like *Quercus faginea* Lam. (*Q. ×tlemcenensis* Trab.) and *Q. pubescens* Willd. (*Q. ×cerrioides* Willk. & Costa) ^14,24,25^, which hampers taxonomic identification and the proper georeferentiation of pure stands dominated by this species.

***Quercus faginea*** Lam. (1785: 725)

The Portuguese oak congregated two widely known subspecies, that were traditionally considered two independent taxa: 1 – *Q. broteroi* and 2 – *Q. faginea* respectively ^15,26-29^. However, the study of the type material of *Q. faginea* carried by Vazquez, et al. ^15^ at the Lamarck herbarium (P-Lam), concluded that the name formerly assigned to the Western and Southern Iberian species as *Q. broteroi* corresponds, in fact, to the own original concept of *Q. faginea* described by Lamarck ^2^*.* This outcome is in accordance with the nomenclatural endorsements given by Schwarz ^30^ and Vasconcellos and Franco ^13^, while later the correct taxonomic and geographic circumscription of the Portuguese Oak is well-stablished by Villar ^31^. This taxon is distributed across the submediterranean and Atlantic areas of the Iberian Peninsula and North Africa (Morocco, Algeria and Tunisia), preferably in base-rich soils, being common in limestone areas. It is the white oak that better stands lower annual precipitations, in stations that are often fed by an increase in summer rainfall ^20,28,32-36^.

***Quercus lusitanica*** Lam. (1785: 719)

The Lusitanian dwarf-oak is a geoxylic shrub, that is exclusive of siliceous bedrock and occurs in the extreme western areas of the Iberian Peninsula, with a sub-population in Morocco. It has a sharp Atlantic distribution, being sensitive to winter cold ^19,37^. It has a past nomenclatural entanglement with *Q. faginea*, since traditional works often mixed up both names and address the name *Q. lusitanica* to the Portuguese oak (*Q. faginea*) ^25,38,39^. The correct nomenclatural endorsement for this shrub was correctly solved and addressed by Sampaio ^40^.

***Quercus boissieri*** Reut. (1853: 119)

The Boissier Oak grows preferably in deep and base-rich soils that are often poor in humus and may vary from sandstones to loam-rich soils. However, it is also compatible with dry and skeletical soils, in places with higher annual rainfall ^41-43^. The name *Q. boissieri* has been traditionally synonymized to the subspecies of *Q. infectoria* (*Q. infectoria* subsp. *veneris* (A.Kern.) Meikle) by several authors and following the priority name proposed by Meikle ^44^. None withstanding, when comparing the type material of *Q. boissieri* available in Geneve Herbarium (G00150110!) to the original plants used for the description of *Q. infectoria* G. Olivier, properly studied in Paris (P00753892!), it became notorious that they should be treated as taxonomically distinct, agreeing with both proposals of Avishai ^9^ and Schwarz ^30^. The presence of totally glabrous leaves and hybrids with *Q. cerris* among the type materials of *Q. infectoria* (P00753892!), are opposed to the lanceolate to oblong leaf blade, with serrate to crenate margin, and the common presence of a flake indumentum observed in the nerves of *Q. boissieri* specimens. This last character gives a bluish tonality to the abaxial leaf blade of the latter species, and the sum of these characters appear as major reliable source to segregate both *taxa*. Simultaneously, this character, led to the proposition of the Series *Orientales* by Schwarz ^30^, where the author congregates *Q. boissieri* and *Q. canariensis* as related species. Avishai ^9^, also segregates *Q. boissieri* by its distribution, mostly through southern and some central parts of Anatolia, Cyprus, Syria, Lebanon, Caucasus and reaching Zagros Mountains in Iran, as farther eastern distribution, normallly in scrub and anthropogenic changed landscapes, up to 1850 m.a.s.l. ^45^. It forms mostly pure stands, or sometimes mixes with other oak species (*Q. brantii, Q. cerris, Q. palaestina, Q. ithaburensis, Q. libani, Q. pubescens*), with direct implication to several suggested hybrids with other oaks ^46,47^. It occupies both Mediterranean and Irano-Turanian regions, and this is reflected in the plasticity of its leaf traits, when changing from mesic to xeric and continental conditions ^47^.

***Quercus infectoria*** G.Olivier (1801: 14-15)

*Q. infectoria* is mainly distributed across north and western Anatolia, from the Aegean to the Black Sea, from which revised materials in Paris Herbarium helped to solve this biogeographic dichotomy against *Q. boisseri* (Paris Herbarium Review – Digital Annex). It can be generally recognized by its shorter petioles, broadly obovate leaves, and prominently crenate or serrate margins, with mostly glabrous leaves, when in contrast to *Q. boisseri*. It occurs often intermixed with *Q. cerris* and *Q. pubescens* stands and sometimes *Pinus brutia* mixed stands, or in pseudo-maquis (*Paliurus sp*., *Arbutus sp*.) up to 850 m, preferably in humus-poor, loamy and base-rich soils ^9^. As it is a Euro-Siberian and temperate element, it mainly distributes across the Euxine and sub-Euxine sectors, being tolerant to winter cold.

**Subsection *Dascia*** (Kotschy) Schwarz ^30^

***Quercus kotschyana*** O.Schwarz (1935: 466)

The Kotschy Oak have traditionally suffered of a long history of taxonomic and nomenclatural misinterpretations, being often ascribed to a taxon resultant from putative introgression with *Q. pubescens,* or *Q. cerris,* and even to a petraeoid oak related to the concepts of *Q. subalpina* O. Schwarz and *Quercus cedrorum* Kotschy ^10,46,48^. Douaihy et al. (2020) conducted a preliminary molecular survey of this species and suggested that together with *Q. vulcanica* Boiss. ex Kotschy, and possibly *Q. macranthera* subsp. *syspirensis* (K.Koch) Menitsky it might represent a member of an ancient stock of closely related, disjoint oaks from section *Quercus*. This entanglement comes from a plethora of multiple interpretation of the morphological plasticity of its leaf blade, expressed by the type population in Bscharri (Lebanon) (Paris Herbarium Review – Digital Annex). This plasticity encompasses the leaf that ranges from a pinnatifid to pinnatilobate margin, with a tomentose leaf blade ^47^. This species has been correctly recovered and solved by Stephan and Teeny ^10^ as an Endangered oak that occurs in fragmented eight sub-populations in Lebanon. The distribution is characterized by isolated trees among *Cedrus libani* mountain forests mixed with other species such as *Quercus petreae subsp. pinnatiloba (Q. cedrorum), Acer hyrcanum tauricolum, Abies cilicica* and *Juniperus spp.* ^49^. The species thrives mainly in cool mesic conditions, and accommodates with different soil conditions but is restricted between 1600 and 2000 m.a.s.l ^50^.

***Quercus pyrenaica* Willd.** (1805: 451)

The Pyrenean oak has a wide distribution, mostly through the submediterranean and temperate belts of the western Mediterranean basin, with important relictual subpopulations in mountain areas of the Mediterranean half of the Iberian Peninsula and North Africa, reaching up to France. It’s a totally edaphic-indifferent species, being more common above siliceous bedrock, and supporting slight summer drought, especially when in comparison to *Q. robur* L ^36,51^.

***Quercus vulcanica*** Boiss. & Heldr. ex Kotschy (1859: 18)

The Kasnak oak is an endemic tree that was originally described from central Anatolia (Karadağ Mountain), which is an extinct volcano located between Konya and Karaman ^45^. Its main distribution range is across the western Central Anatolia to the upper Mediterranean Turkey region (known as Turkish Lakes), in a Natural Conservation area for the species. Some few isolated populations have also been recorded towards the east in Kayseri (Ahır and Gediz Mountains), Hatay (Amanos and Murat Mountains), Ankara (Sarıçalı Mountain) and towards the north in the Western Black Sea Region of Turkey ^11,52-54^. It prefers mesophytic conditions, ocurring on limestone bedrock, basalt rocks, volcanic rocks, and ophiolite, normally as pure stands or intermixed with *Pinus* spp.*, Cedrus libani, Acer hyrcanum,* and other oaks (*Q. infectoria, Q. pubescens, Q. cerris, Q. petraea, Q. robur, Q. trojana*) from 1100-2000 m ^11,55^. Kasnak oak differs from *Q.petraea subsp. pinnatiloba* (K.Koch) Menitsky by having flat cup scales, and leaves with truncated lobes and intercalary veins, and from *Q. frainetto* Ten. by having longer petioles and the leaves evenly distributed across the shoots ^45^.

**Supplementary Table S1**

Supplementary Table S4 - Evaluation scores per species for the ensemble of best models (TSS: True skill statistic; AUC: Area Under the Curve; Sensitivity also called true positive rate or recall; Specificity also defined as the true negative rate)

| **Region** | **Species** | **TSS** | **AUC** | **Sensitivity** | **Specificity** |
| --- | --- | --- | --- | --- | --- |
| Eastern Mediterranean | *Quercus kotschyana* | 1,00 | 1,00 | 100,00 | 100,00 |
| Western Mediterranean | *Quercus lusitanica* | 0,96 | 1,00 | 100,00 | 96,42 |
| Eastern Mediterranean | *Quercus vulcanica* | 0,91 | 0,99 | 97,67 | 93,29 |
| Western Mediterranean | *Quercus canariensis* | 0,90 | 0,99 | 96,43 | 93,52 |
| Western Mediterranean | *Quercus pyrenaica* | 0,90 | 0,99 | 96,65 | 92,79 |
| Eastern Mediterranean | *Quercus boissieri* | 0,88 | 0,99 | 97,02 | 90,48 |
| Eastern Mediterranean | *Quercus infectoria* | 0,84 | 0,97 | 97,39 | 86,16 |
| Western Mediterranean | *Quercus faginea* | 0,82 | 0,97 | 93,32 | 88,90 |

**Supplementary Table S2 – Species Records (Digital Annex)**

**Supplementary Table S3**

Supplementary Table S3 – Species record numbers.

| **Sub-Region** | **Species** | **Number of records** |
| --- | --- | --- |
| Western Mediterranean | *Q. pyrenaica* | 8689 |
| Western Mediterranean | *Q. faginea* | 1321 |
| Western Mediterranean | *Q. lusitanica* | 613 |
| Western Mediterranean | *Q. canariensis* | 165 |
| Eastern Mediterranean | *Q. boissieri* | 1470 |
| Eastern Mediterranean | *Q. vulcanica* | 154 |
| Eastern Mediterranean | *Q. infectoria* | 118 |
| Eastern Mediterranean | *Q. kotschyana* | 15 |

**Supplementary Table S4**

Supplementary Table S4 - Models’ summary for past, current and future projections.

| **Projections** | **Model** | **Code** | **Time period** |
| --- | --- | --- | --- |
| Past | Heinrich Stadial 1 | HS | 17.0-14.7 Kyr |
|  | Bølling-Allerød | BA | 14.7-12.9 Kyr |
|  | Younger Dryas Stadial | YDS | 12.9-11.7 Kyr |
|  | Early-Holocene | EH | 11.7-8.3 Kyr |
|  | Mid-Holocene | MH | 8.3-4.2 Kyr |
|  | Late-Holocene | LH | 4.2-0.3 Kyr |
| Current | Average model of 5 GCMs | Current | 1981-2010 |
| Future | Average model of 5 GCMs × 3 SSP (126, 370, 585) | sspXX_2070 | 2070 |
|  |  | sspXX_2100 | 2100 |

Supplementary Figure S1. - Ensemble response curves showing the relationship between climatic suitability and the four predictors retained after multicollinearity filtering (BIO02, BIO06, BIO18 and BIO19). Temperature-related predictors (BIO02 and BIO06) are displayed on the left, while precipitation-related predictors (BIO18 and BIO19) are shown on the right. Curves represent marginal responses derived from ensemble species distribution models, with species grouped by eastern and western Mediterranean regions.

**Supplementary Figure S2**

Supplementary Figure S2. - Mean (± SD) contribution of the four climatic predictors (BIO02, BIO06, BIO18 and BIO19) across species and regions. Bars represent mean permutation-based variable importance across ensemble models, with error bars indicating standard deviation among algorithms. Values are shown separately for eastern and western Mediterranean species groups.

**Supplementary Figure S3**

Supplementary Figure S3 - Past latitude (a) and longitude (b) changes by species and period. Positive increases in latitude represent northern-driven shifts, while for longitude, these are eastern-driven shifts.

**Supplementary Figure S4**

Supplementary Figure S4 - Past loss (%) by species and period.

**Supplementary Figure S5**

Supplementary Figure S5 - Future loss (%) by species and period.

**Supplementary Figure S6**

Supplementary Figure S6 - a) Past vs. future latitude change (%) for all species / periods combinations; b) Past vs. future latitude change (%) by species group (East vs West).

**Supplementary Figure S7**

Supplementary Figure S7 - a) Past vs. future latitude change (%) for all species by past period; b) Past vs. future latitude change (%) for all species by future period scenario/year.

**Supplementary Figure S8**

Supplementary Figure S8 - a) Past vs. future longitude change (%) for all species/periods combinations; b) Past vs. future longitude change (%) by species group (east vs west).

**Supplementary Figure S9**

Supplementary Figure S9 - a) Past vs. future Longitude change (%) for all species by past period; b) Past vs. future Longitude change (%) for all species by future period scenario/year.

**Supplementary Figure S10**

Supplementary Figure S10 - Past vs. future total change (%) for all species and period combinations.

**Supplementary Figure S11**

Supplementary Figure S11 – a) Past vs. future Loss change (%) for all species/periods combinations; b) Past vs. future Loss change (%) by species group (East vs West).

**Supplementary Figure S12**

Supplementary Figure S12 - a) Past vs. future Loss change (%) for all species by past period; b) Past vs. future Loss change (%) for all species by future period scenario/year.

**Supplementary Figure S13.**

Supplementary Figure S13- Species-specific occurrence maps for the eight circummediterranean marcescent oaks included in this study, compiled from curated field observations, taxonomically validated herbarium records and quality-filtered GBIF data. Panels are ordered from left to right and top to bottom as follows: **(a)** *Quercus canariensis*, **(b)** *Quercus boissieri*, **(c)** *Quercus faginea*, **(d)** *Quercus infectoria*, **(e)** *Quercus pyrenaica*, **(f)** *Quercus vulcanica*, **(g)** *Quercus lusitanica* and **(h)** *Quercus kotschyana*. These maps provide a visual assessment of the geographic distribution, data coverage and sampling consistency of the occurrence records used for SDM calibration, complementing Supplementary Tables S2–S3 and the Digital Annex dataset.

**Species Records and Supplementary Maps – Digital Annex** are available at: <https://zenodo.org/records/15497712?preview=1&token=eyJhbGciOiJIUzUxMiJ9.eyJpZCI6IjM1NjAzYmVjLWFkZjUtNDUyMS05YWMxLTM0ODA1NjU0ZmJlMyIsImRhdGEiOnt9LCJyYW5kb20iOiJjODg0Zjk0YTQxZjNmOWM2MGEwMDEwZWZhYWY1MzQ1NiJ9.pnJd6zIPRIqaxoubXw7tdX546UYQR64jEonS3kiQYi6NMT4RVXS9C16cLwN0UMVhdlzhhTBTyo5vYIzLrxO8uw> DOI: **10.5281/zenodo.15497712**

1	Thiers, B. (New York Botanical Garden's Virtual Herbarium, 2022).

2	Lamarck, J. B. *Encyclopédie méthodique. Botanique*. (Panckoucke, 1785).

3	Willdenow, C. L. *Enumeratio plantarum Horti Regii Berolinensis*. (Taberna libraria Scholae Realis, 1809).

4	Willdenow, C. L. *Species plantarum exhibentes plantas rite cognitas ad genera relatas cum differentiis specificis, nominibus trivialibus, synonymis selectis, locis naturalibus secundum systema sexuale digestas*. Vol. 4 (Nauck, 1805).

5	Olivier, G. A. *Voyage dans l'empire Othoman*. 561 (Henri Agasse, 1807).

6	Boissier, E. *Diagnoses plantarum orientalium novarum.... Ser. 1: Vol. secundum: Fasc. No. 12*. (Wolfrath, 1853).

7	Kotschy, T. *Die Eichen Europa‘s und des Orient‘s*. (E. Hölzel, 1862).

8	Schwarz, O. Einige neue Eichen des Mediterrangebiets und Vorderasiens. *Notizblatt des Königl. botanischen Gartens und Museums zu Berlin* **114**, 461-469, doi:10.2307/3994977 (1935).

9	Avishai, M. *Taxonomic-Geographic Studies in the Middle East Oaks* Master thesis, The Hebrew University of Jerusalem, (1967).

10	Stephan, J. & Teeny, P. Revealing the taxonomy of an endemic oak of Lebanon. *Pl Sociol* **54**, 97-100 (2017).

11	Öney, S. B., Nihal, Ö., Gugger, P. F. & Özkan, K. Endemik kasnak meşesinin (Quercus vulcanica [Boiss. et Heldr. ex] Kotschy) yeni bir yayılış alanı. *Turkish Journal of Forestry* **21**, 6-14 (2020).

12	Yücedağ, C., Müller, M. & Gailing, O. Morphological and genetic variation in natural populations of Quercus vulcanica and Q. frainetto. *Plant Systematics and Evolution* **307**, 1-15 (2021).

13	Vasconcellos, J. C. & Franco, J. A. Os Carvalhos de Portugal. *Anais do Instituto Superior de Agronomia* **21**, 1–135 (1954).

14	Vázquez, F. M. *et al.* Anotaciones a la nomenclatura del género Quercus L., (FAGACEAE) en la Península Ibérica y NW de África. . *Folia Botánica Extremadurensis* **12**, 5-79 (2018).

15	Vazquez, F. M. *et al.* Lectotypification of names of Quercus spp.(Fagaceae) described by Lamarck from the Iberian Peninsula. *Phytotaxa* **455**, 205-213 (2020).

16	Denk, T., Grimm, G. W., Manos, P. S., Deng, M. & Hipp, A. An updated infrageneric classification of the oaks: review of previous taxonomic schemes and synthesis of evolutionary patterns. *Oaks physiological ecology. Exploring the functional diversity of genus Quercus L.*, 13-38 (2017).

17	Spach, É. *Histoire naturelle des végétaux: Phanérogames. Vol. 11. Librairie encyclopédique de Roret, Paris*. (1842).

18	Gürke, M. in *Plantae Europaeae [Enumeratio systematica et synonymica plantarum phanerogamicarum in Europa sponte crescentium vel mere inquilinarum]* Vol. 2 (ed K. Richter) 54–72 (Engelmann, 1897).

19	Vila-Viçosa, C. *et al.* Late Quaternary range shifts of marcescent oaks unveil the dynamics of a major biogeographic transition in southern Europe. *Scientific reports* **10**, 1-12 (2020).

20	Vila-Viçosa, C. *et al.* Combining Satellite Remote Sensing and Climate Data in Species Distribution Models to Improve the Conservation of Iberian White Oaks (Quercus L.). *ISPRS International Journal of Geo-Information* **9**, 735 (2020).

21	Vila-Viçosa, C. *et al.* Syntaxonomic update on the relict groves of Mirbeck's oak (Quercus canariensis Willd. and Q. marianica C. Vicioso) in southern Iberia. *Plant Biosystems* **149**, 512-526, doi:10.1080/11263504.2015.1040484 (2015).

22	Carapeto, A., Francisco, A., Pereira, P. & Porto, M. (Sociedade Portuguesa de Botânica, Associação Portuguesa de Ciência da …, 2020).

23	Carrero, C. *et al.* The red list of oaks 2020. *The Morton Arboretum: Lisle, IL, USA*, 11-12 (2020).

24	Battandier, J. A. & Trabut, L. C. *Flore analytique et synoptique de l'Algérie et de la Tunisie.* . (Vve Giralt, Imprimeur-Éditeur, 1905).

25	Vicioso, C. *Revisión del género Quercus en España*. (Tipografía Artítica, 1950).

26	Rivas-Martínez, S. & Saénz, C. Enumeración de los Quercus de la Península Ibérica. *Rivasgodaya* **6**, 101-110 (1991).

27	Rivas-Martínez, S. *et al.* Vascular plant communities of Spain and Portugal: addenda to the syntaxonomical checklist of 2001. Part II. *Itinera geobotanica*, 433-922 (2002).

28	Vila-Viçosa, C., Vázquez F.M., Meireles C & Pinto-Gomes, C. Taxonomic peculiarities of marcescent oaks (Quercus, Fagaceae) in southern Portugal. *Lazaroa* **35**, 139–153, doi:10.5209/rev_LAZA.2014.v35.42555 (2014).

29	Vila-Viçosa, C. *Os carvalhais marcescentes do Centro e Sul de Portugal. Estudo e Conservação* MSc thesis, Évora, (2012).

30	Schwarz, O. Entwurf zu einem natürlichen System der Cupuliferen und der Gattung Quercus L. *Notizblatt des Botanischen Gartens und Museums zu Berlin-Dahlem*, 1-22 (1936).

31	Villar, E. H. Estudios sobre los Quercus del Oeste mediterráneo. *Anales del Instituto Botanico de A.J. Cavanilles* **15**, 3-114 (1958).

32	Meddour, R., Meddour-Sahar, O., Zeraia, L. & Mucina, L. Syntaxonomic synopsis of the forest and tall scrub vegetation of Northern Algeria. *Lazaroa* **38**, 127-163 (2017).

33	Babali, B., Hasnaoui, A., Medjati, N. & Bouazza, M. Note on the vegetation of the mounts of tlemcen (Western Algeria): Floristic and phytoecological aspects. *Open Journal of Ecology* **3**, 370 (2013).

34	Taleb, M. S. & Fennane, M. *Vascular plant communities of Morocco*. (Springer, 2019).

35	Vila-Viçosa, C. *et al.* Temporihygrophilous Quercus broteroi forests in southern Portugal: analysis and conservation. *Plant Biosystems* **146**, 298-308, doi:10.1080/11263504.2012.678 (2012).

36	Blanco-Castro, E. *et al.* *Los bosques ibéricos. Una interpretación geobotánica*. 597 (Editorial Planeta, Barcelona, Spain, 2005).

37	Capelo, J. H., Costa, J. C., Lousã, M. F. & Mesquita, S. A aliança Quercion fruticosae Rothmaler 1954 em. Rivas-Martínez, Lousã, T.E. Díaz, Fernández-González & J.C. Costa 1990. *Quercetea* **3**, 99-110 (2002).

38	Webb, P. *Iter Hispaniense.*, (Béthune and Plon, 1838).

39	Coutinho, A. X. P. Os Quercus de Portugal. *Boletim da Sociedade Broteriana* **6**, 47-116 (1888).

40	Sampaio, G. Nota a propósito dos Quercus lusitanica Lamk., Q. humilis Lamk e Quercus faginea, Lamk. . *Annaes Scientificos da Academia Polytechnica do Porto* **5**, 161-165 (1910).

41	Ugurlu, E. & Senol, S. G. Quercus-dominated vegetation units in the Aegean region of Turkey. *Botanika Chronika* **18**, 283-291 (2005).

42	Ketenoglu, O. *et al.* Synopsis of syntaxonomy of Turkish forests. *Journal of Environmental Biology* **31**, 71 (2010).

43	Ghazal, A. Landscape ecological, phytosociological and geobotanical study of eumediterranean in west of Syria. (2008).

44	Meikle, R. D. *Flora of Cyprus 2*. (Royal Botanic Gardens, Kew, 1985).

45	Hedge, I. & Yaltırık, F. Quercus L. *Flora of Turkey and the East Aegean Islands* **7**, 659-683 (1982).

46	Douaihy, B. *et al.* Tracking diversity and evolutionary pathways of Lebanese oak taxa through plastome analyses. *Botany Letters* **167**, 315-330 (2020).

47	Stephan, J. M., Teeny, P. W., Vessella, F. & Schirone, B. Oak morphological traits: between taxa and environmental variability. *Flora* **243**, 32-44 (2018).

48	Jablonski, E. Karl Georg Theodor Kotschy and the Kotschy Oak. (2016). <<https://www.internationaloaksociety.org/content/karl-georg-theodor-kotschy-and-kotschy-oak> >.

49	Stephan, J. Quercus kotschyana. The IUCN Red List of Threatened Species 2018:. (2018). <<https://www.iucnredlist.org/species/194180/2303139> >.

50	Stephan, J., Chayban, L. & Vessella, F. Abiotic factors affecting the distribution of oaks in Lebanon. *Turkish Journal of Botany* **40**, 595-609 (2016).

51	Vilches, B., Sánchez-Mata, D. & Gavilán, R. G. in *Vegetation Structure and Function at Multiple Spatial, Temporal and Conceptual Scales*	257-283 (Springer, 2016).

52	Yaltırık, F. *Türkiye’nin Meşeleri Teşhis Kılavuzu*. (1984).

53	Avcı, M. Endemik bir meşe türü, kasnak meşesi (Quercus vulcanica (Boiss. Heldr. ex) Kotschy)'nin Türkiye'deki yeni bir yayılış alanı. *Türk Coğrafya Dergisi*, 283-289 (1996).

54	Aydınözü, D. Kasnak meşesi (quercus vulcanica (boiss. and heldr. ex) kotschy)’nin Türkiye’deki ikinci yeni bir yayılış alanı. (2004).

55	Kargıoğlu, M., Şenkul, Ç., Serteser, A. & Konuk, M. Bioclimatic requirements of Quercus vulcanica Boiss et Heldr. ex Kotschy an endemic species in Turkey. *Polish Journal of Ecology* **57**, 197-200 (2009).
